# How to experimentally evaluate the adiabatic condition for quantum annealing

**DOI:** 10.1038/s41598-024-58286-2

**Published:** 2024-04-08

**Authors:** Yuichiro Mori, Shiro Kawabata, Yuichiro Matsuzaki

**Affiliations:** 1 Global Research and Development Center for Business by Quantum-AI Technology (G-QuAT), National Institute of Advanced Industrial Science and Technology (AIST), 1-1-1, Umezono, Tsukuba, Ibaraki 305-8568 Japan; 2 NEC-AIST Quantum Technology Cooperative Research Laboratory, National Institute of Advanced Industrial Science and Technology (AIST), Tsukuba, Ibaraki 305-8568 Japan

**Keywords:** Quantum information, Qubits, Optical spectroscopy

## Abstract

We propose an experimental method for evaluating the adiabatic condition during quantum annealing (QA), which will be essential for solving practical problems. The adiabatic condition consists of the transition matrix element and the energy gap, and our method simultaneously provides information about these components without diagonalizing the Hamiltonian. The key idea is to measure the power spectrum of a time domain signal by adding an oscillating field during QA, and we can estimate the values of the transition matrix element and energy gap from the measurement output. Our results provides a powerful experimental basis for analyzing the performance of QA.

## Introduction

The adiabatic theorem is a crucial result in quantum mechanics, first introduced by Ehrenfest in 1916^1^. Later, Born and Fock proved a more modern version of the theorem in 1928^2^. The theorem states that if an initial state is prepared in the ground state of the Hamiltonian, it will remain in the ground state as long as the change in the Hamiltonian is slow enough. Since Born and Fock’s proof in 1928, there have been numerous studies that have improved and expanded the theorem, including more rigorous formulations^3^ and extensions to open systems^4–6^.

An essential application of this theorem is quantum annealing (QA). This was originally proposed by Apolloni *et al*. in^7^. The original proposal aimed to improve the simulated annealing utilizing the quantum effects of tunneling. However, an alternative approach was subsequently presented^8,9^, where the Hamiltonian changes over time. In this approach, a ground state of the transverse-field Hamiltonian is prepared, and the Hamiltonian is gradually changed to the target problem Hamiltonian. The adiabatic theorem guarantees that if the alteration of the Hamiltonian is gradual enough, the final state will be the ground state of the problem Hamiltonian.

QA has been intensively studied from various viewpoints, including the computational speed^10–12^, implementation methods^13,14^, and algorithms^15–17^. The commercial use of QA machines was pioneered by D-Wave Systems Inc. Accordingly, proposals for their use in research and applications in various fields have arisen, including examples in quantum chemistry^18,19^, machine learning^20,21^, and high-energy physics^22^. For more information, see review papers^23–25^.

One of the problems in QA is that there is no known efficient method for checking whether the adiabaticity is satisfied or not. In principle, if we can diagonalize the Hamiltonian, we can use an approximate version of the adiabatic conditions are given as follows^23,24,26,27^:

1 $$\begin{aligned} \frac{|{\langle {m(s)|\dot{\mathcal {H}}(s)|0(s)}\rangle }|}{|E_{m}(s)-E_{0}(s)|^{2}}\ll T_{\rm ann} \end{aligned}$$

for all *s* and *m*, where $T_{\rm ann}$ denotes the annealing time, $s=t/T_{\rm ann}$ denotes the time normalized by $T_{\rm ann}$, *t* denotes the time, ${|{m(s)}\rangle }$ (${|{0(s)}\rangle }$) denotes the *m*-th excited (ground) state, $\dot{\mathcal {H}}(s)$ denotes the *s* derivative of the instantaneous Hamiltonian at a time *s* and $E_m(s)$ ($E_0(s)$) denote the eigenenergies of the *m*-th excited (ground) state (see Supplemental Material). Throughout this paper, we consider a dimensionless time *s* normalized by $T_{\rm ann}$. These conditions are obtained by an argument that considers only the first order perturbation expansion and neglects higher order terms^28^, and so are not mathematically rigorous. In particular, conditions (1) are not known to be sufficient for adiabaticity. However, when the interest is in the qualitative properties of the computation time, these conditions are widely used, and so we adopt them as the adiabatic conditions in our paper.

In the case of applying QA to practical problems, it is unworkable to diagonalize the Hamiltonian with using a classical computer. Consequently, we cannot directly apply the adidabatic conditions (1) to check whether the dynamics is adiabatic or not. Experimental methods have been proposed to measure the energy gap^17,29,30^, which corresponds to the denominator in Eq. (1). However, to our knowledge, no studies have been conducted to measure the numerator of the adiabatic condition (1), *i.e.*, the size of the transition matrix element of the time derivative of the Hamiltonian.

In this paper, we propose a method for simultaneously measuring the numerator and denominator of Eq. (1). This method involves utilizing an oscillating field during quantum annealing to induce a Rabi oscillation between the ground and excited states. By performing Fourier transformation on a time domain signal, we obtain a power spectrum and extract relevant information from the data. These steps enable us to evaluate the values of the numerator and denominator of the adiabatic condition (1).

The remainder of this paper is organized as follows. In Sect. "Review of QA", we review QA. In Sect."Our method for evaluating the adiabatic condition", we introduce our method for simultaneously measuring the values of the transition matrix element and the energy gap, based on an analytical calculation using some approximations. In Sect. "Numerical analysis", we describe numerical simulations (with noise) performed to quantify the performance of our method in realistic cases. Lastly, we summarize our results and discuss possible directions for open questions.

## Review of QA

To review the conventional QA, we consider the following Hamiltonian:

2 $$\begin{aligned} \mathcal {H}_{{\rm conv}}(s) =f(s) \mathcal {H}_{{\rm D}} + (1-f(s)) \mathcal {H}_{{\rm P}}, \end{aligned}$$

where $\mathcal {H}_{{\rm D}}$ is a driver Hamiltonian, $\mathcal {H}_{{\rm P}}$ is a problem Hamiltonian, and *f*(*s*) is a schedule function satisfying the condition

3 $$\begin{aligned} f(0) = 1,\ f(1) = 0. \end{aligned}$$

Here and in the following we make the choice

4 $$\begin{aligned} f(s)&=1-s. \end{aligned}$$

Due to the condition (3), the Hamiltonian at $s=0$ is the driver Hamiltonian $\mathcal {H}_{{\rm D}}$ and the Hamiltonian at $s= 1$ is the problem Hamiltonian. After obtaining a ground state of the driver Hamiltonian, we let the state evolve by the annealing Hamiltonian from $s=0$ to $s=1$. According to the adiabatic theorem, if the annealing time $T_{\rm ann}$ is sufficiently large, the state after QA becomes a ground state of the problem Hamiltonian.

## Our method for evaluating the adiabatic condition

We will now present a technique to experimentally determine the numerator and denominator of the left-hand side of Eq. (1) for a given time $s_{1}$, using the Hamiltonian defined in Eq. (2). In this scenario, the Hamiltonian in Eq. (1) is the Hamiltonian for quantum annealing $\mathcal {H}_{{\rm conv}}$ defined by Eq. (2). We introduce the Hamiltonian $\mathcal {H}(s)$, which comprises the driver Hamiltonian $\mathcal {H}_{\rm D}$, the problem Hamiltonian $\mathcal {H}_{\rm P}$, and an external driving Hamiltonian $\mathcal {H}_{\rm ext}(s)$ with strength $\lambda (s)$ and frequency $\omega$ as follows.

5 $$\begin{aligned} \mathcal {H}(s)&=\mathcal {H}_\mathrm{{QA}}(s) +\mathcal {H}_{{\rm ext}}(s) \end{aligned}$$

6 $$\begin{aligned} \mathcal {H}_\mathrm{{QA}}(s)&= A(s) \mathcal {H}_{{\rm D}} + (1-A(s)) \mathcal {H}_{{\rm P}} \end{aligned}$$

7 $$\begin{aligned} \mathcal {H}_{{\rm ext}}(s)&= \lambda (s) \dot{\mathcal {H}}_\mathrm{{conv}}(s_{1})\cos \left( \omega T_{\rm ann} (s-s_1)\right) \end{aligned}$$

**Figure 1:**
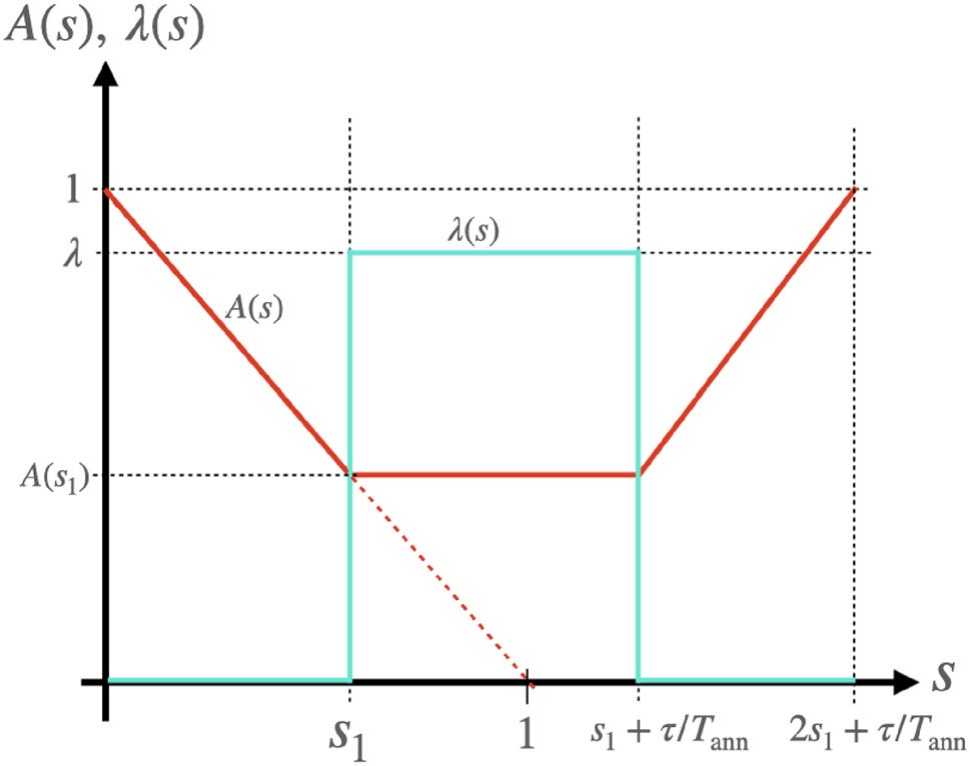
Plot of the scheduling function *A*(*t*) and strength of the external driving field $\lambda$ for our protocol. The dotted line shows *f*(*s*), which is the scheduling function of the conventional QA.

Here, *A*(*s*) is the schedule function that modulates the weight of $\mathcal {H}_{\rm D}$ and $\mathcal {H}_{\rm P}$ in $\mathcal {H}_{\rm QA}(s)$. We plot *A*(*s*) and $\lambda (s)$ as functions of time in Fig. 1, where we note that *A*(*s*) satisfies $A(s)=f(s)$ when $0\le s\le s_{1}$.

Our experimental protocol proceeds as follows. Firstly, we prepare the ground state of the driver Hamiltonian ${|{0(s=0)}\rangle }$. Secondly, we slowly vary the Hamiltonian $H_{\rm QA}(s)$ from $s=0$ to $s=s_{1}$ by setting $\lambda (s) = 0$, allowing the system to evolve under this Hamiltonian adiabatically. Thirdly, at $s=s_{1}$, we introduce a driving term by setting $\lambda (s) = \lambda$ and fixing $A(s)=f(s_{1})$, and we let the system evolve for $s_{1}<s\le s_{1}+\tau /T_{\rm ann}$. Fourthly, we terminate the driving at $s=s_{1}+\tau /T_{\rm ann}$ by setting $\lambda (s) = 0$, and gradually vary the Hamiltonian from $H_{\rm QA}(s_{1})$ to $H_{\rm D}$ for $s_{1}+\tau /T_{\rm ann} <s\le 2s_{1}+\tau /T_{\rm ann}$, allowing the system to evolve adiabatically. Finally, we measure the probability of the system occupying the *m*-th excited state ${|{m(s=0)}\rangle }$ of the driver Hamiltonian using projective measurements, which we denote as $p_{0,m}(\omega , s_1, \tau )$. We repeat these steps multiple times, varying $\omega , s_{1}$ and $\tau$. We emphasize the importance of adiabaticity during the second and fourth steps, while it is not necessary for the third step.

In the method described above, we assume that the adiabaticity is satisfied from $s=0$ to $s=s_1$, and we will discuss how this assumption could be roughly justified in a realistic circumstance in the concluding Section.

Let us explain how to realize $\mathcal {H}_{{\rm ext}}(s)$ in the third step of the actual experiment. We have

8 $$\begin{aligned} \mathcal {H}_{{\rm ext}}(s)&= \lambda \dot{f}(s_1) \mathcal {H}_{{\rm D}} \cos \left( \omega T_{\rm ann} (s-s_1)\right) \nonumber \\ & \quad-\lambda \dot{f}(s_1) \mathcal {H}_{{\rm P}} \cos \left( \omega T_{\rm ann}(s-s_1)\right) . \end{aligned}$$

In the experiment with superconducting qubits, we can temporarily change the coefficient of the Pauli matrices^31,32^. The driver Hamiltonian and problem Hamiltonian can be decomposed using the Pauli operators as follows:

9 $$\begin{aligned} \mathcal {H}_{{\rm D}}&=\sum _i h_{i}\mathcal {O}_{i}, \end{aligned}$$

10 $$\begin{aligned} \mathcal {H}_{{\rm P}}&=\sum _j h'_{j}\mathcal {O}'_{j}, \end{aligned}$$

where $\mathcal {O}_{i}$ ($\mathcal {O}'_{j}$) denote the Pauli matrices and $h_{i}$ ($h'_{j}$) denotes a time-independent coefficient. Hence, we obtain

11 $$\begin{aligned} \mathcal {H}_{{\rm ext}}(s)=\sum _i \lambda \dot{f}(s_1) h_i \mathcal {O}_{i} \cos \left( \omega T_{\rm ann} (s-s_1)\right) \nonumber \\ -\sum _{j'} \lambda \dot{f}(s_1) h_j' \mathcal {O}_{j'} \cos \left( \omega T_{\rm ann}(s-s_1)\right) . \end{aligned}$$

Thus, if we can temporarily change the coefficient of the Pauli matrices to a cosine function, it is possible to realize the Hamiltonian $\mathcal {H}_{{\rm ext}}(s)$.

As the problem Hamiltonian usually contains two-body interaction terms, we must change the interaction coupling strength. Such a technique has also been developed for superconducting circuits^33^.

Here, we describe the dynamics of the system in the third step of our scheme, which is crucial for measuring the adiabatic condition. We begin by describing a simplified scenario in which the dynamics is adiabatic in the second and fourth steps, and we will consider more general cases later. For simplicity, we omit the expression of “$(s_{1})$” to mention $\mathcal {H}_{\rm QA}(s_{1})$ or $\dot{\mathcal {H}}_{\rm conv}(s_{1})$ in the remainder of this section. In our proposal, the measurements are performed while sweeping the time period $\tau$; hence, we treat $\tau$ as a variable in the remainder of this section unless mentioned otherwise.

Let us diagonalize $\mathcal {H}_{{\rm QA}}$ as follows:

12 $$\begin{aligned} \mathcal {H}_{{\rm QA}} = \sum _{i}E_{i}{|{i}\rangle }{\langle {i}|}, \end{aligned}$$

where $E_i\le E_j$ is satisfied for $i < j$. By moving to a rotating frame, we can express the state of the system as follows:

13 $$\begin{aligned} {|{\tilde{\psi }(\tau )}\rangle }=e^{ir\tau \mathcal {H}_{{\rm QA}}}{|{\psi (\tau )}\rangle }, \end{aligned}$$

and the Hamiltonian in the rotating frame is expressed as

14 $$\begin{aligned} \mathcal {\tilde{H}}(\tau )&= e^{ir\tau \mathcal {H}_{{\rm QA}}}\mathcal {H}(\tau )e^{-ir\tau \mathcal {H}_{{\rm QA}}}+i\frac{d e^{ir\tau \mathcal {H}_{{\rm QA}}}}{d\tau }e^{-ir\tau \mathcal {H}_{{\rm QA}}}\nonumber \\&=(1-r)\mathcal {H}_{{\rm QA}} + e^{ir\tau \mathcal {H}_{{\rm QA}}}\mathcal {H}_{{\rm ext}}(\tau )e^{-ir\tau \mathcal {H}_{{\rm QA}}}. \end{aligned}$$

Note that we set $\hbar = 1$ throughout this paper. Here, we assume that the transition frequency between the ground state and the *m*-th excited state is close to the frequency of the external driving field. Then, we set *r* as the ratio between $|E_{m}-E_{0}|$ and $\omega$ as follows:

15 $$\begin{aligned} r=\frac{\omega }{|E_{m}-E_{0}|}, \end{aligned}$$

where $E_{0}$ denotes the energy of the ground state. The second term in Eq. (14) becomes

16 $$\begin{aligned} & e^{{ir\tau {\mathcal{H}}_{{{\text{QA}}}} }} {\mathcal{H}}_{{{\text{ext}}}} (\tau )e^{{ - ir\tau {\mathcal{H}}_{{{\text{QA}}}} }} \\ & \quad \quad = \lambda \sum\limits_{{i,j}} {\left\langle {i\left| {{\dot{\mathcal{H}}}_{{{\text{conv}}}} } \right|j} \right\rangle } e^{{ir(E_{i} - E_{j} )\tau }} \cos \omega \tau \left| i \right\rangle \left\langle j \right|. \\ \end{aligned}$$

Here, we adopt the rotating wave approximation (RWA)^34^. The coefficient ${|{i}\rangle }{\langle {j}|}$ in Eq. (16) includes an oscillatory component:

17 $$\begin{aligned} & e^{{ir(E_{i} - E_{j} )\tau }} \cos \omega \tau \\ & \quad = \frac{1}{2}e^{{ir(E_{i} - E_{j} )\tau }} (e^{{i\omega \tau }} + e^{{ - i\omega \tau }} ) \\ & \quad = \frac{1}{2}e^{{i(r(E_{i} - E_{j} ) - \omega )\tau }} + \frac{1}{2}e^{{i(r(E_{i} - E_{j} ) + \omega )\tau }} . \\ \end{aligned}$$

If $r|E_{i}-E_{j}|= \omega$ is satisfied, one of the terms in Eq. (17) becomes time-independent while the other term has a high-frequency oscillation. Owing to the condition of Eq. (15), we have at least two time-independent terms, $(i,j) = (m,0)$ and (0, *m*), which remain after RWA. We assume a condition $||E_{m}-E_{0}|-\omega |\ll ||E_{i}-E_{j}|-\omega |$ in neither $(i,j)= (m,0)$ nor $(i,j) = (0, m)$. Then, all terms except $(i,j) = (0,m)$ and $(i,j)=(m,0)$ are dropped, and the Hamiltonian (16) can be simplified as $\mathcal {H}_{{\rm ext},I}=\frac{\lambda }{2}{\langle {m|\dot{\mathcal {H}}_{\rm conv}|0}\rangle }{|{m}\rangle }{\langle {0}|} + h.c.$. Therefore, the effective Hamiltonian Eq. (14) can be expressed as

18 $$\begin{aligned} \mathcal {H}_{{\rm eff}}= \sum _{i} (1-r)E_{i}{|{i}\rangle }{\langle {i}|}+\frac{\lambda }{2}{\langle {m|\dot{\mathcal {H}}_{\rm conv}|0}\rangle }{|{m}\rangle }{\langle {0}|} + h.c.. \end{aligned}$$

These calculations indicate that if the initial state is prepared in a subspace spanned by the ground state and *m*-th excited state, the system’s dynamics will be confined to this subspace. Notably, projecting out the states except ${|{m(s=s_{1})}\rangle }$ and ${|{0(s=s_{1})}\rangle }$ results in an effective Hamiltonian with the same structure as the single-qubit Hamiltonian that induces Rabi oscillations. A known analytical formula that characterizes tha Rabi oscillation without decoherence involves two parameters: detuning and Rabi frequancy, and details of the behavior of Rabi oscillations in a single-qubit system are presented in Supplemental Material. By using this analytical formula, we can fit the data obtained from our method and acquire information about the transition matrix element $|{\langle {m|\dot{\mathcal {H}}|0}\rangle }|$ and the energy gap $E_{\rm m}-E_{0}$.

To observe the oscillation experimentally, we need to construct a projective measurement of ${|{m}\rangle }{\langle {m}|}$ in the rotating frame. In our idea, the fourth and fifth steps enable us to construct a projective measurement ${|{m}\rangle }{\langle {m}|}$ in the laboratory frame effectively, provided the dynamics in the fourth step is adiabatic. If the state ${|{\psi (\tau )}\rangle }$ is an eigenstate of the Hamiltonian $\mathcal {H}_{\rm QA}$, the change in the frame only results in a global phase. Therefore, as long as the second step and fourth step are adiabatically performed, $p_{0,m}(\omega , s_1, \tau )$ is approximately described as follows:

19 $$\begin{aligned} p_{0,m}(\omega , s_1, \tau )&\simeq |{\langle {m|e^{-i\tau \mathcal {H}_{{\rm eff}}}|0}\rangle }|^{2} \nonumber \\&\propto (1-\cos \Omega _{{\rm ana}}(\omega )\tau ), \end{aligned}$$

where $\Omega _{{\rm ana}}(\omega )$ is analytically expected angular frequency of the Rabi oscillation given by,

20 $$\begin{aligned} \Omega _{{\rm ana}}(\omega ) = \sqrt{\left( \lambda |{\langle {m|\dot{\mathcal {H}}_{\rm conv}|0}\rangle }|\right) ^{2}+(\omega -\Delta )^{2}}, \end{aligned}$$

where $\Delta = E_{m} - E_{0}$. We obtain the right-hand side of Eq. (19), which is independent of $s_1$, under an assumption that the adiabatic condition is satisfied at the second and fourth steps. However, if there are non-adiabatic transitions, the probability $p_{0,m}$ has a dependence on $s_1$.

In the aforementioned discussion, a ground state of the driver Hamiltonian is assumed to be prepared in the first step, and we perform a projective measurement into the *m*-th excited state in the fifth step. Meanwhile, if we prepare the *k*-th excited state in the first step and perform a projective measurement into the *l*-th excited state in the fifth step, we can obtain the angular frequency of Rabi oscillation as $\Omega _{{\rm ana}}^{(k,l)}(\omega ) = \sqrt{\left( \lambda |{\langle {l|\dot{\mathcal {H}}_{\rm conv}|k}\rangle }|\right) ^{2}+(\omega -\Delta _{kl})^{2}}$, where $\Delta _{kl}=E_{k} - E_{l}$ through similar calculations. The details of these derivations are presented in Supplemental Material. When non-adiabatic transition is caused, the signal may include some different oscillations, whose angular frequencies are given by $\Omega _{{\rm ana}}^{(k,l)}(\omega )$.

The adiabatic condition described in Eq. (1) is valid only when we can consider that the effect of the non-adiabatic transitions is weak. Therefore, throughout our paper, we assume that the effect of non-adiabatic transitions is negligible. We will discuss how the non-adiabatic transitions affect the spectroscopic measurements in our methods later.

Let us explain how to specify the values of $|E_m-E_0|$ and $|{\langle {m|\dot{\mathcal {H}}_{\rm conv}|0}\rangle }|$ by using our method. We repeat these by sweeping $\omega$, and we can find an optimal value of $\omega =|E_m-E_0|$ to minimize the frequency of the Rabi oscillation; this corresponds to the energy gap $\Delta$. Furthermore, the Rabi frequency with the optimal $\Omega$ observed in our method corresponds to the numerator in Eq. (1). Thus, our estimated transition matrix element $|{\langle {m|\dot{\mathcal {H}}|0}\rangle }|_{{\rm est}}$ and our estimated energy gap $\Delta _{{\rm est}}$ are given by

21 $$\begin{aligned}{} & {} \lambda |{\langle {m|\dot{\mathcal {H}}_{\rm conv}|0}\rangle }|_{{\rm est}}&=\min _{\omega }[\Omega _{{\rm exp}}(\omega )], \end{aligned}$$

22 $$\begin{aligned}{} & {} \Delta _{{\rm est}}&=\mathop {\text {arg~min}}\limits _{\omega }\left[ \Omega _{{\rm exp}}(\omega )\right] , \end{aligned}$$

respectively. Here, $\Omega _{{\rm exp}}(\omega )$ is the angular frequency of the Rabi oscillation obtained experimentally, which is analytically considered to be expressed by Eq. (20).

In actual experiments, owing to some imperfections, $p_{0,m}(\omega , t_{1}, \tau )$ cannot be fully explained by the analytical formula Eq. (19), which was derived under ideal conditions (see Fig. 1 in the Supplemental Materials). To find the relevant frequency of $\Omega _{{\rm exp}}(\omega )$ in the dynamics, we perform a Fourier transformation and obtain a power spectrum that is defined by

23 $$\begin{aligned} P(\omega ,s_{1}, \Omega )&= \textrm{abs}\left[ \textrm{FT}[p_{0,m}(\omega , s_{1}, \tau )]\right] ,\nonumber \\&=\textrm{abs}\left[ \int _{-\infty }^{\infty }d\tau \ p_{0,m}(\omega ,s_{1},\tau )\frac{e^{-i\Omega \tau }}{\sqrt{2\pi }}\right] . \end{aligned}$$

If $p_{0,m}(\omega ,s_{1},\tau )$ is expressed as Eq. (19), the power spectrum is given by

24 $$\begin{aligned} P(\omega ,s_{1},\Omega ) =&\alpha (\omega )\delta (\Omega ) + \frac{\alpha (\omega )}{2}\delta (\Omega -\Omega _{{\rm ana}}(\omega ))\nonumber \\&+\frac{\alpha (\omega )}{2}\delta (\Omega +\Omega _{{\rm ana}}(\omega )). \end{aligned}$$

Therefore, in the actual experiment, we define the peak with a positive frequency in the spectrum as $\Omega _{{\rm exp}}(\omega )$, and we expect to satisfy $\Omega _{{\rm exp}}(\omega ) \simeq \Omega _{{\rm ana}}(\omega )$ in the power spectrum; this allows us to use the formulas of Eqs. (21) and (22). Thus, we can estimate the values of the transition matrix element $|{\langle {m|\dot{\mathcal {H}}_{\rm conv}|0}\rangle }|$ and the energy gap $\Delta$ using our method.

Lastly, we discuss potential experimental implementations of our proposal. In the existing D-wave quantum annealing plattform, it is not possible to perform our proposal because we cannot perform microwave pulses to induce the Rabi oscillation in the annealer. However, the recently proposed method, called the spin-lock quantum annealing^13,35^, is compatible with the requirement of our method. In the spin-lock quantum annealing, we use superconducting qubits for a gate type quantum computer, and there is an experimental demonstration of the spin-lock with superconducting qubits by using microwave pulses^36^.

## Numerical analysis

We perform numerical simulations to evaluate the effectiveness of our method. In the previous section, we derived an analytical formula under the following assumptions:

- I. The time evolution is adiabatic in both step 2 and step 4.
- II. The rotating wave approximation holds.
- III. The time evolution in step 3 only involves the ground state and the *m*-th excited state.
- IV. There is no decoherence.

However, these assumptions may not be met in actual experiments and we perform numerical simulations to examine the validity of our method under different conditions, as summarized in Table 1.

**Table 1:** Cases investigated in this study. For cases B, C, E, and F, we consider the effect of non-adiabatic transitions in steps 2 and 4. Meanwhile, for cases C and F, we consider decoherence.

Case	Qubit	Adiabaticity of	Decoherence	Violated
	Number	Step 2 and 4		conditions
A	1	Complete	None	II
B	1	Incomplete	None	I, II
C	1	Incomplete	$\checkmark$	I, II, IV
D	2	Complete	None	II, III
E	2	Incomplete	None	I, II, III
F	2	Incomplete	$\checkmark$	I, II, III, IV

Condition I is only satisfied when the process in steps 2 and 4 is completely adiabatic. In cases A and D from Table 1, we use diagonalization to prepare the ground state of $\mathcal {H}_{{\rm QA}}(s_{1})$. In the remaining cases, we solve the time-dependent Schrödinger equation with specific annealing times to prepare the ground state of $\mathcal {H}_{{\rm QA}}(s_{1})$.

Condition III is naturally satisfied for a single-qubit system, while it is violated for a system with two or more qubits. Thus, in cases A, B, and C, condition III is satisfied, whereas in cases D, E, and F, condition III is violated.

Condition IV is satisfied if we solve a time-dependent Schrödinger equation of the system, as with cases A, B, D, and E. Meanwhile, we consider the effect of decoherence by solving the master equation in cases C and F.

### Settings and methods for all cases

Here, we introduce some conditions that are common throughout our numerical analysis.

#### Schedule function

For the schedule function *A*(*s*) in Eq. (5), we use

25 $$\begin{aligned} A(s) = {\left\{ \begin{array}{ll} 1-s&{}\ (0\le s< s_{1}),\\ 1-s_{1}&{}\ (s_{1}\le s< s_{1}+\frac{\tau }{T_{\rm ann}}),\\ s-2s_{1}-\frac{\tau }{T_{\rm ann}}&{}\ (s_{1}+\frac{\tau }{T_{\rm ann}}\le s < 2s_{1}+\frac{\tau }{T_{\rm ann}}), \end{array}\right. } \end{aligned}$$

where $T_{{\rm ann}}$ is the annealing time. In actual experiments, this value is typically around 10 to 100 $\mu$s, and the typical energy scale of the Hamiltonian is of the order of GHz in the superconducting qubits (*e.g.* D-wave system^37^). We choose smaller values $T_{\rm ann}$ such as 10 to 1000 ns in the following simulations to consider worse cases where the non-adiabatic transitions could occur.

We take the schedule function (25) as $A(s) = 1-s$ up to $s_1$, and we evaluate the adiabatic condition at time $s_{1}$ according to our method. Hence, for our simulation, $\dot{\mathcal {H}}_\mathrm{{QA}}(s_{1})$ is given by

26 $$\begin{aligned} \dot{\mathcal {H}}_{\rm conv}(s_{1}) =- \mathcal {H}_{{\rm D}} + \mathcal {H}_{{\rm P}}, \end{aligned}$$

for any $$t_{1}$$.

#### Strength $\lambda$

The Rabi frequency can be controlled by changing the strength $\lambda$. If the decoherence is negligible, we set $\lambda$ to be as small as possible, because RWA is valid only when the Rabi frequency is much smaller than the energy gap. Meanwhile, when there is decoherence, the choice of $\lambda$ is not straightforward. As we decrease $\lambda$, the decoherence becomes more relevant and RWA becomes more valid. Therefore, the following condition should be satisfied:

27 $$\begin{aligned} {\frac{1}{T_{c}}\ll \lambda |{\langle {m|\dot{\mathcal {H}}_{\rm conv}|0}\rangle }|\ll |E_{1}-E_{0}|}, \end{aligned}$$

where $T_{c}$ is the coherence time. In our simulation, we set $\lambda = 0.05$, chosen as a value that satisfies the condition of Eq. (27) in our numerical simulations.

#### Time evolution and measurement process

In real experiments, we need to consider decoherence. To address this, we use the Gorini–Kossakowski–Sudarshan–Lindblad (GKSL) master equation^38^,

28 $$\begin{aligned} \dot{\rho } = -i[\mathcal {H},\rho ] + \sum _{n} (L_{n}\rho L_{n}^{\dag } - \frac{1}{2}\{L_{n}^{\dag }L_{n},\rho \}), \end{aligned}$$

for cases C and F, where $L_{n}$ is the Lindblad operators.

**Figure 2:**
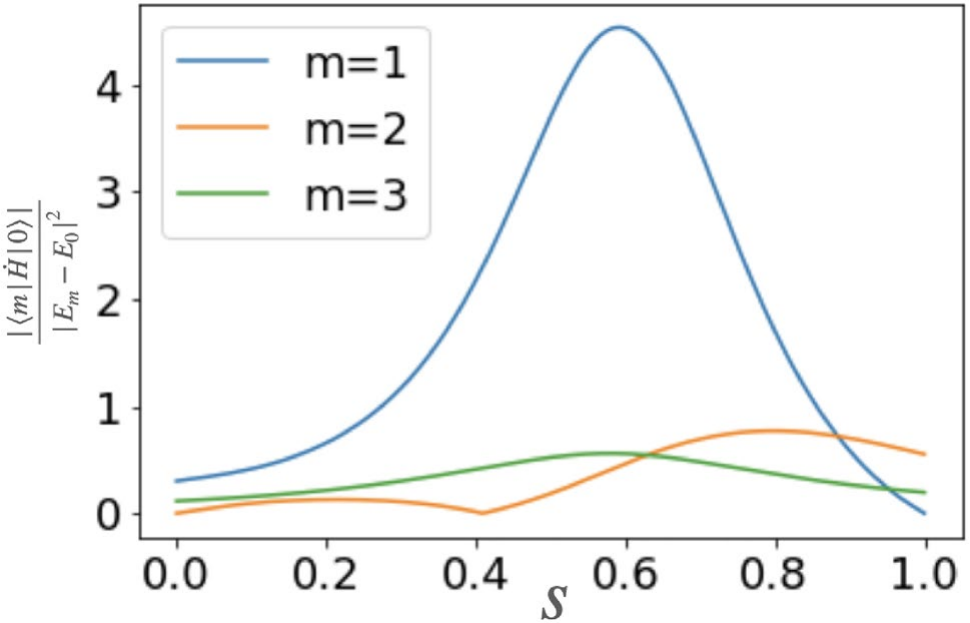
The actual adiabatic conditions (1) of our simulated two-qubit cases. $m=1$ is the largest at almost all *s*.

In the fifth step, we assume that an ideal projective measurement into the state ${|{m(t=0)}\rangle }$ can be performed. It is worth mentioning that, in the actual experiment, this projective measurement corresponds to $\sigma _{x}$ on all qubits. By using a post processing with a classical computer, we can obtain the projection probability for not only $m=1$ but also all *m*. However, the non-adiabatic transitions between the ground state and the first excited state is considered as the most relevant part. Actually, as long as $|{\langle {1|\dot{H}|0}\rangle }|$ is similar to or larger than $|{\langle {m|\dot{H}|0}\rangle }|$ for $m\ge 2$, the non-adiabatic transitions between the ground state and the first excited state is more relevant than the others, Thus, for the numerical simulations, we consider a case of $m=1$ in this paper. (See Fig. 2)

#### Construction of $\Omega _{{\rm exp}}(\omega )$

In our method, we calculate the probability of a projection into the first excited state $p_{0,1}(\tau )$, and we use the power spectrum $P(\Omega )$ to determine the function $\Omega _{{\rm exp}}(\omega )$ as explained in the previous section. In this case, we expect to observe a peak at $\Omega =\Omega _{{\rm ana}}(\omega )$ in the power spectrum. To determine the function $\Omega _{{\rm exp}}(\omega )$, we fix $\omega$ and maximize the height of the power spectrum by sweeping $\Omega$ so that we can determine the position of the resonance peak as follows:

29 $$\begin{aligned} \Omega _{{\rm exp}}(\omega ) = \mathop {\text {arg~max}}\limits _{\Omega } P(\Omega , \omega ). \end{aligned}$$

Finally, by sweeping $\omega$, we can obtain the function $\Omega _{{\rm exp}}(\omega )$.

When we sweep $\Omega$, it is crucial to choose an appropriate range. First, we explore the frequency range $\Omega >0$. As indicated by Eq. (24), three peaks emerge. However, to evaluate the adiabatic condition, our focus lies solely on the positive-frequency peak, because the negative frequency peak contains the same information as its positive counterpart, while the zero frequency peak lacks relevant information. Also, we should consider only the frequency range of $\Omega \ll \omega$ because we use RWA to derive the analytical formula of Eq. (19), which is valid only for $\Omega \ll \omega$.

Even if we restrict the frequency range, we may not find a correct peak for several reasons. We discuss the case in which such a problem occurs, and we present a possible solution to overcome such a problem at least for some cases.

### Single-qubit cases (A, B, and C)

**Figure 3:**
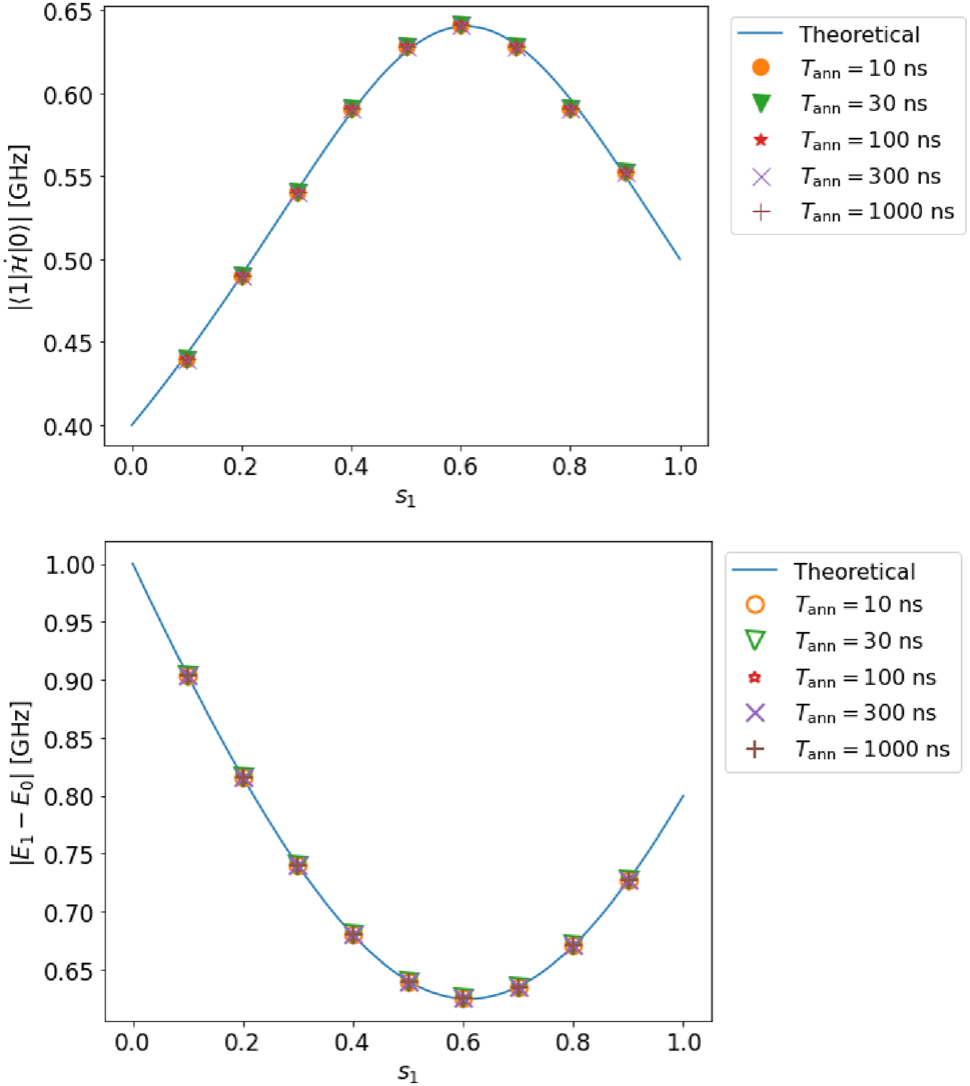
(Top) Estimation of the transition matrix element in case A (single qubit, complete adiabaticity, and no decoherence). (Bottom) Estimation of the energy gap in case A. The solid lines represent the solution obtained by diagonalization of the Hamiltonian, and the dots represent the estimated values obtained from our method by numerical simulation.

We examine the single-qubit cases (A, B, and C). For these cases, the driver Hamiltonian $\mathcal {H}_{{\rm D}}$ and the problem Hamiltonian $\mathcal {H}_{{\rm P}}$ are given by

30 $$\begin{aligned} \mathcal {H}_{{\rm D}} = \frac{\omega _{1}}{2}\sigma _{x},\quad \mathcal {H}_{{\rm P}} =g\sigma _{z}, \end{aligned}$$

respectively. In our simulation, we fixed $\omega _{1}=1\ \textrm{GHz}$ and $g = 0.4\ \textrm{GHz}$.

#### Case A

We set the parameters $T_{\rm ann}$ and $s_{1}$ as follows:

31 $$\begin{aligned} T_{{\rm ann}}&=10,~ 30,~ 100,~ 300,~ 1000\ \textrm{ns},\nonumber \\ s_{1}&= 0.1,~ 0.2,~ 0.3,~ 0.4,~ ..., 0.9. \end{aligned}$$

As shown in Fig. 3, our estimated values (dots in the figure) are in good agreement with the theoretically expected values (lines in the figure). Indeed, the relative error in the estimation of the transition matrix element $|{\langle {1|\dot{\mathcal {H}}|0}\rangle }|$ (the energy gap $E_{1}-E_{0}$) is at most $0.99~\%$ ($0.071~\%$).

These errors are small compared to the resolution owing to the discretization performed while processing the data. The estimation error of the transition matrix element (energy gap) is 0.9 (0.1) times smaller than the resolution. As shown in Fig. 3, we confirm that the adiabatic condition (1) is reasonably satisfied.

#### Case B

**Figure 4:**
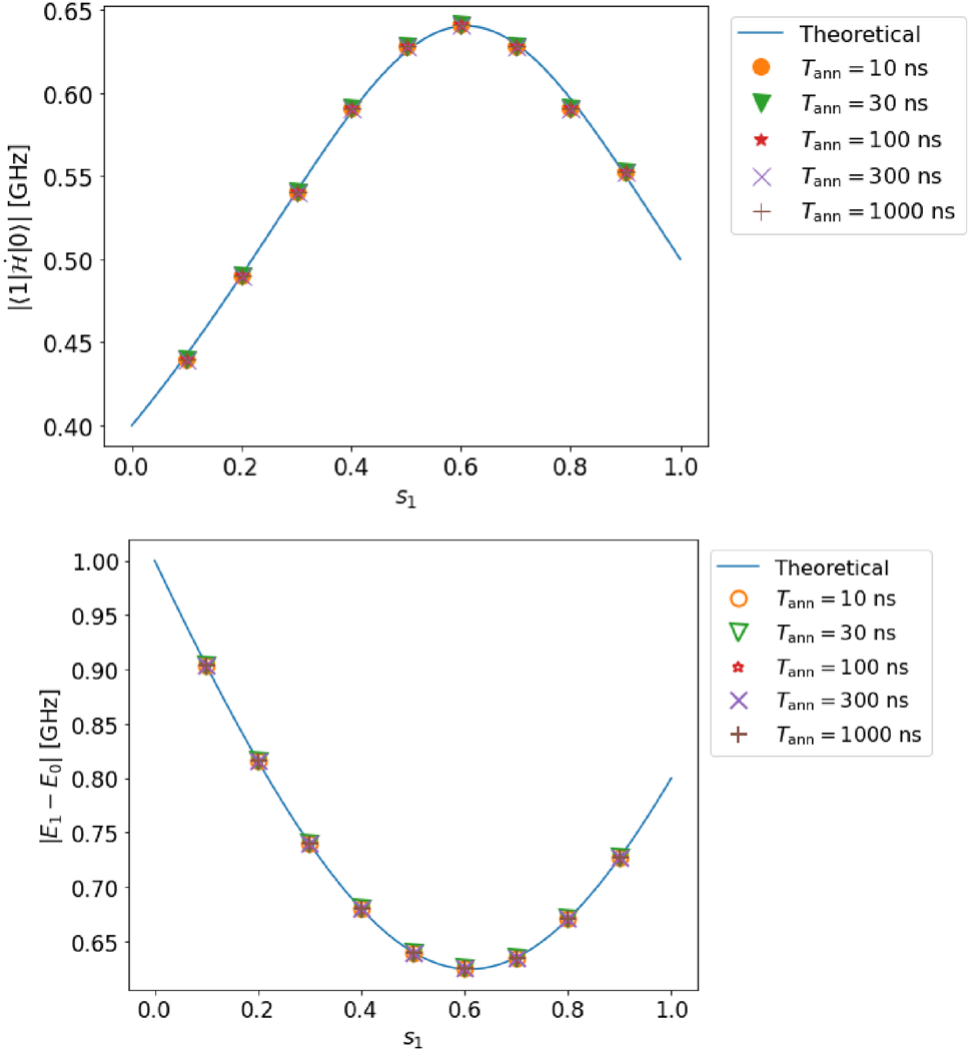
Top (bottom): estimated value of the transition matrix element (energy gap) in case B (single qubit, incomplete adiabaticity, and no decoherence). For the solid lines and dots, we use the same notation as that in Fig. 3.

**Figure 5:**
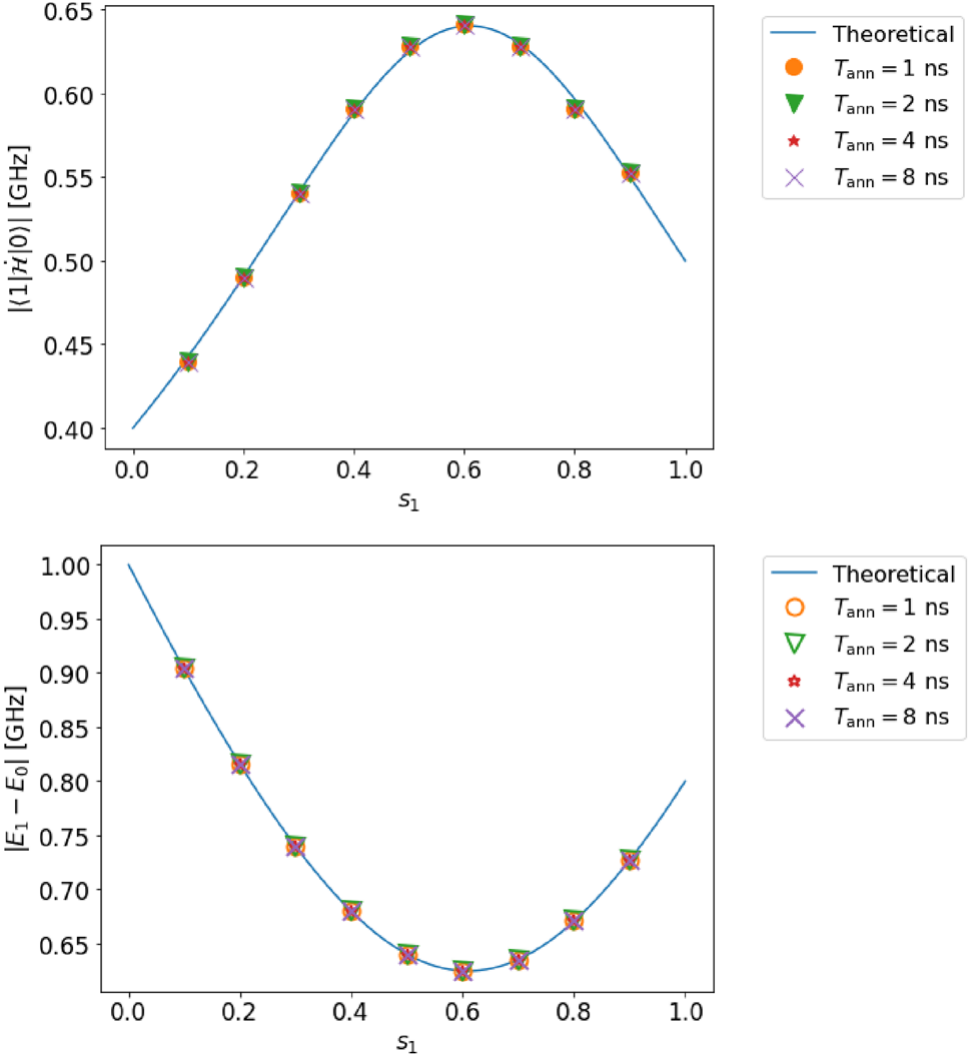
Top (bottom): estimated value of the transition matrix element (energy gap) in case B (single qubit, incomplete adiabaticity, and no decoherence) with a shorter annealing time such as $T_{{{{\rm ann}}}} = 1,2,4,8$ $\textrm{ns}$. For the solid lines and dots, we use the same notation as that in Fig. 3.

Next, the effect of non-adiabatic transitions in steps 2 and 4 is studied for case B. Similar to case A, we can accurately measure both the transition matrix element $|{\langle {1|\dot{\mathcal {H}}|0}\rangle }|$ and the energy gap $(E_{1}-E_{0})$ for case B, and (see Fig. 4) the relative error of the transition matrix element (energy gap) is at most $2.2~\%$ ($0.7~\%$) and 0.77 (0.93) relative to the resolution.

For the single-qubit case, our scheme is robust against the non-adiabatic transitions. Actually, we consider cases with $T_{\rm ann}=1, 2, 4$, and 8 $\textrm{ns}$ (see Fig. 5), and these results show that a shorter annealing time does not impair the performance of our methods.

We show that, as long as RWA is valid, the power spectrum contains a peak corresponding to a frequency of $\Omega (\omega )$ (see Supplemental Material). Thus, we can accurately estimate the transition matrix element and energy gap using Eqs. (21) and (22) for the single-qubit case without decoherence.

#### Case C

**Figure 6:**
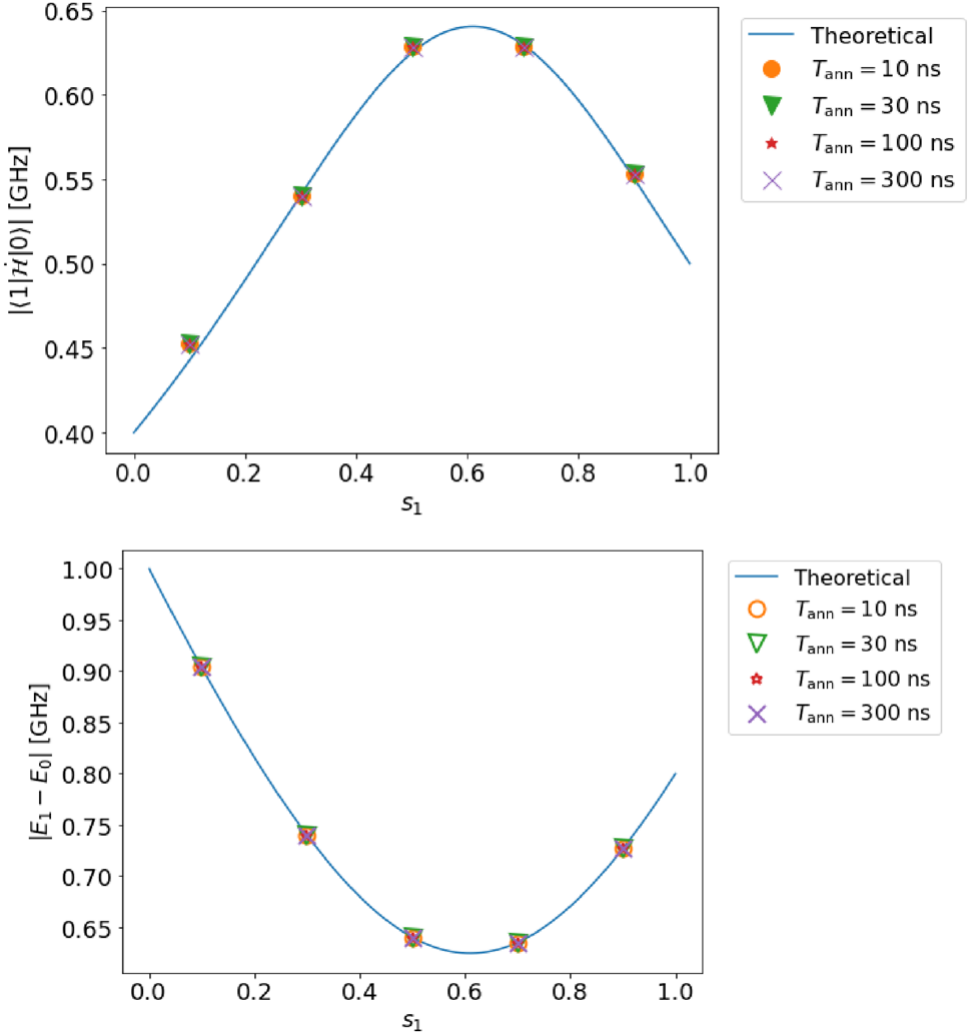
Top (bottom): estimated value of the transition matrix element (energy gap) in case C (single qubit, incomplete adiabaticity, and decoherence). For the solid lines and dots, we use the same notation as that in Fig. 3.

In case C, to consider decoherence, we employ the GKSL master equation, and we select the Lindblad operator as

32 $$\begin{aligned} L=\sqrt{\kappa } \sigma _{z}, \end{aligned}$$

where $\kappa$ denotes the decay rate. We fix $\kappa =2.5\times 10^{-3}$ $\mathrm {ns^{-1}}$, which is a typical value for a superconducting flux qubit^39^

The results are shown in Fig. 6. The relative error of the transition matrix element $|{\langle {1|\dot{\mathcal {H}}|0}\rangle }|$ (the energy gap $\Delta$) is at most $2.1~\%$ ($0.05~\%$), which is 0.77 (0.023) times smaller than the resolution.

These errors are as small as those in cases A and B, indicating the robustness of our method against decoherence. This resilience stems from the fact that decoherence primarily impacts the width rather than the position of the peaks in the power spectrum. Consequently, accurate estimation of the transition matrix element and energy gap remains achievable even in the presence of weak decoherence.

### Two-qubit cases (D, E, and F)

**Figure 7:**
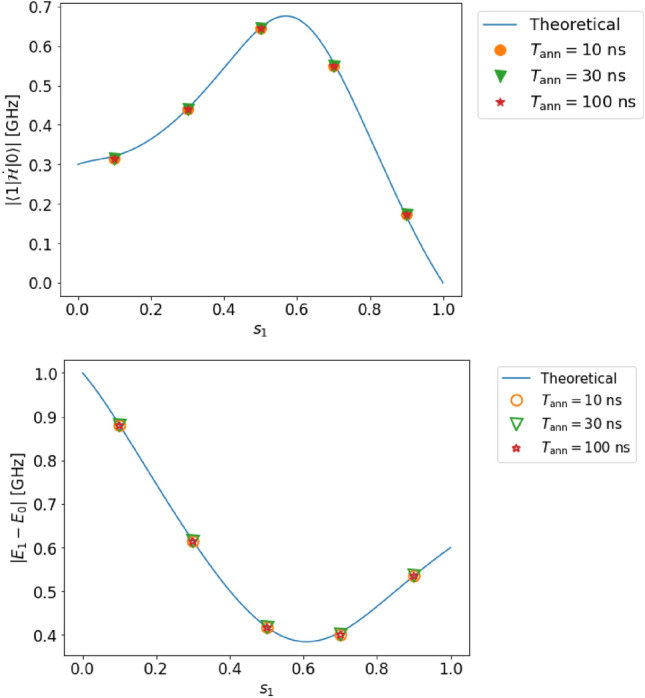
Top (bottom): estimated value of the transition matrix element (energy gap) in case D (two qubits, complete adiabaticity, and no decoherence). Even when two qubits are used, we can estimate both the transition matrix element and the energy gap with high accuracy. For the solid lines and dots, we use the same notation as that in Fig. 3.

In the two-qubit cases, the problem and driver Hamiltonians are given by

33 $$\begin{aligned} \mathcal {H}_{{\rm D}}&= \frac{\omega _{1}}{2}\sigma _{x}\otimes 1 + \frac{\omega _{2}}{2}1\otimes \sigma _{x},\nonumber \\ \mathcal {H}_{{\rm P}}&=g_{1}\sigma _{z}\otimes \sigma _{z} + g_{2}\sigma _{z}\otimes 1 + g_{3}1\otimes \sigma _{z}, \end{aligned}$$

respectively. Here, we set $\omega _{1} = 1.0\ \textrm{GHz}$, $\omega _{2} = 1.1\ \textrm{GHz}$, $g_{1} = 0.5\ \textrm{GHz}$, $g_{2}=0.3\ \textrm{GHz}$, and $g_{3}=0$.

For these cases, we select the parameters $T_{\rm ann}$ and $s_{1}$ as follows.

34 $$\begin{aligned} T_{{\rm ann}}&=10,~ 30,~ 100\ \textrm{ns,}\nonumber \\ s_{1}&= 0.1,~ 0.3,~ 0.5,~ 0.7,~ 0.9. \end{aligned}$$

#### Case D

In this case, we can accurately measure the transition matrix element $|{\langle {1|\dot{\mathcal {H}}|0}\rangle }|$ and the energy gap $(E_{1}-E_{0})$ as shown in Fig. 7. The relative error of the transition matrix element (energy gap) is at most $3.5~\%$ ($0.04~\%$), which is 0.55 (0.99) times smaller than the resolution. Despite not satisfying condition III for considering two qubits in this case, the dynamics can be effectively confined within a two-level system, ensuring the accuracy of our method, especially when the Rabi frequency is low.

#### Case E

**Figure 8:**
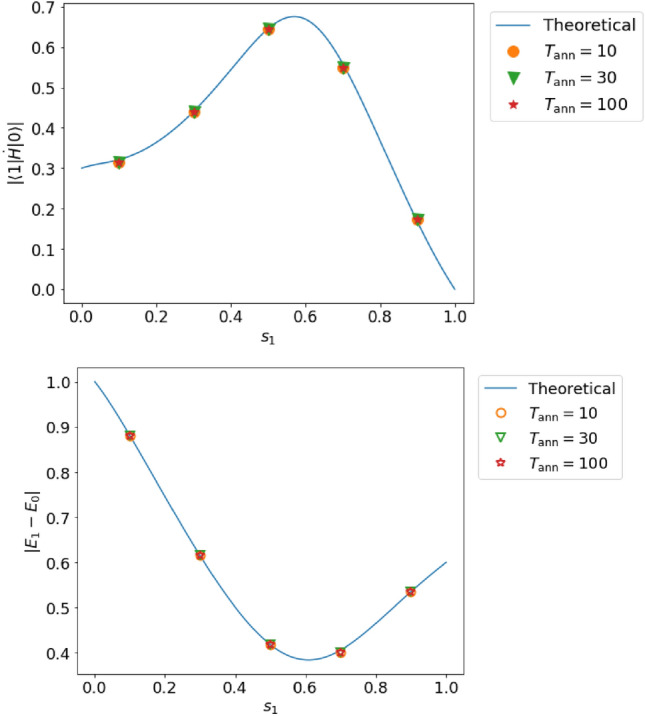
Top (bottom): estimated value of the transition matrix element (energy gap) in case E (two qubits, incomplete adiabaticity, and no decoherence). For the solid lines and dots, we use the same notation as that in Fig. 3.

In case E, the relative error of the transition matrix element (energy gap) is at most $3.5~\%$ ($1.2~\%$), which is 0.55 (0.99) times smaller than the resolution, as shown in Fig. 8.

In the case of weak non-adiabatic transitions, it is possible to estimate both the transition matrix element and the energy gap with high accuracy even for the two-qubit case. Meanwhile, as described in detail in Supplemental Material, in the case of strong non-adiabatic transitions, the power spectrum contains peaks other than the one that we want to use in our estimation. We discuss a possible solution for this problem in Suppremental Material.

#### Case F

**Figure 9:**
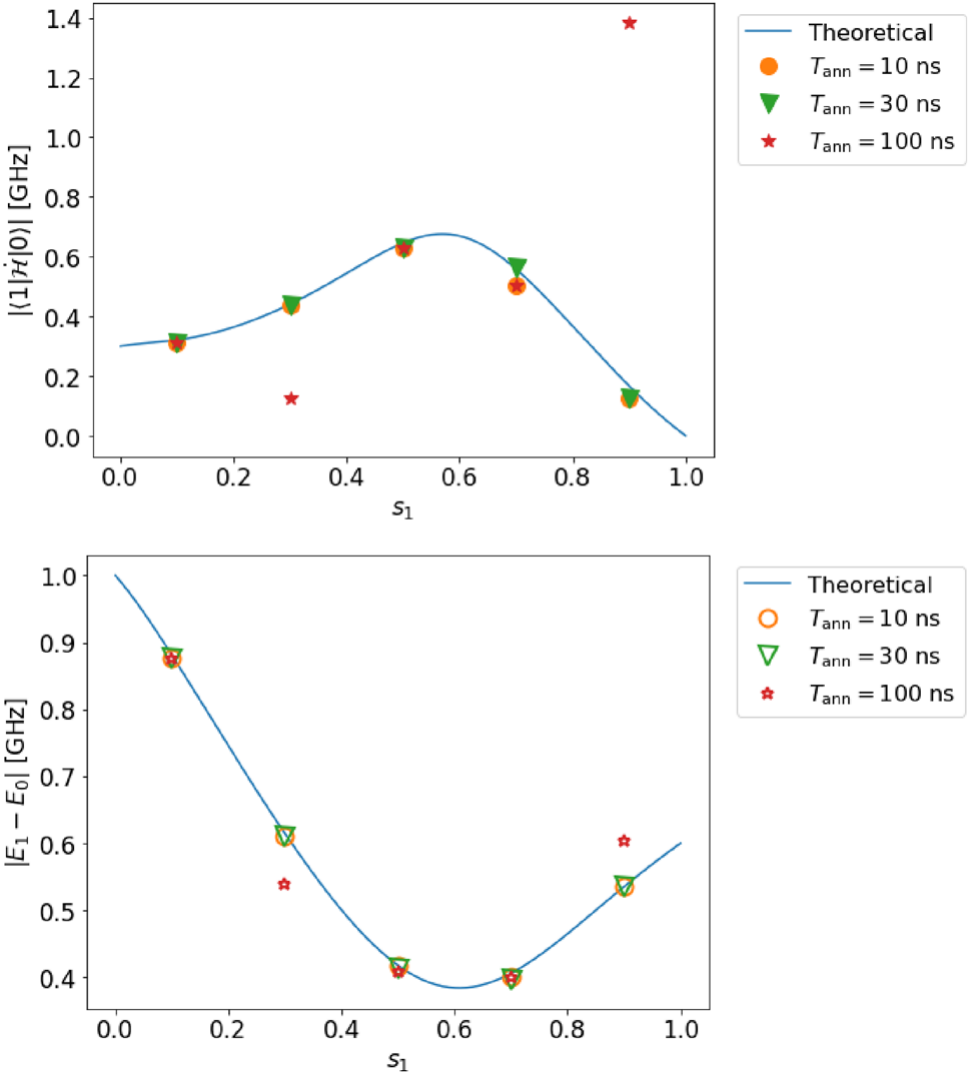
Top (bottom): estimated value of the transition matrix element (energy gap) in case F (two qubits, incomplete adiabaticity, and decoherence). In this case, we have significant estimation errors for a few points. For the solid lines and dots, we use the same notation as that in Fig. 3.

In case F, we select the Lindblad operator as follows.

35 $$\begin{aligned} L_{1} =\sqrt{\kappa }\sigma _{z}\otimes 1,\qquad L_{2} = \sqrt{\kappa } 1\otimes \sigma _{z}, \end{aligned}$$

Here, $\kappa$ denotes the decay rate. For the numerical simulations, we chose $\kappa =2.5\times 10^{-3}$ $\textrm{ns}^{-1}$.

**Figure 10:**
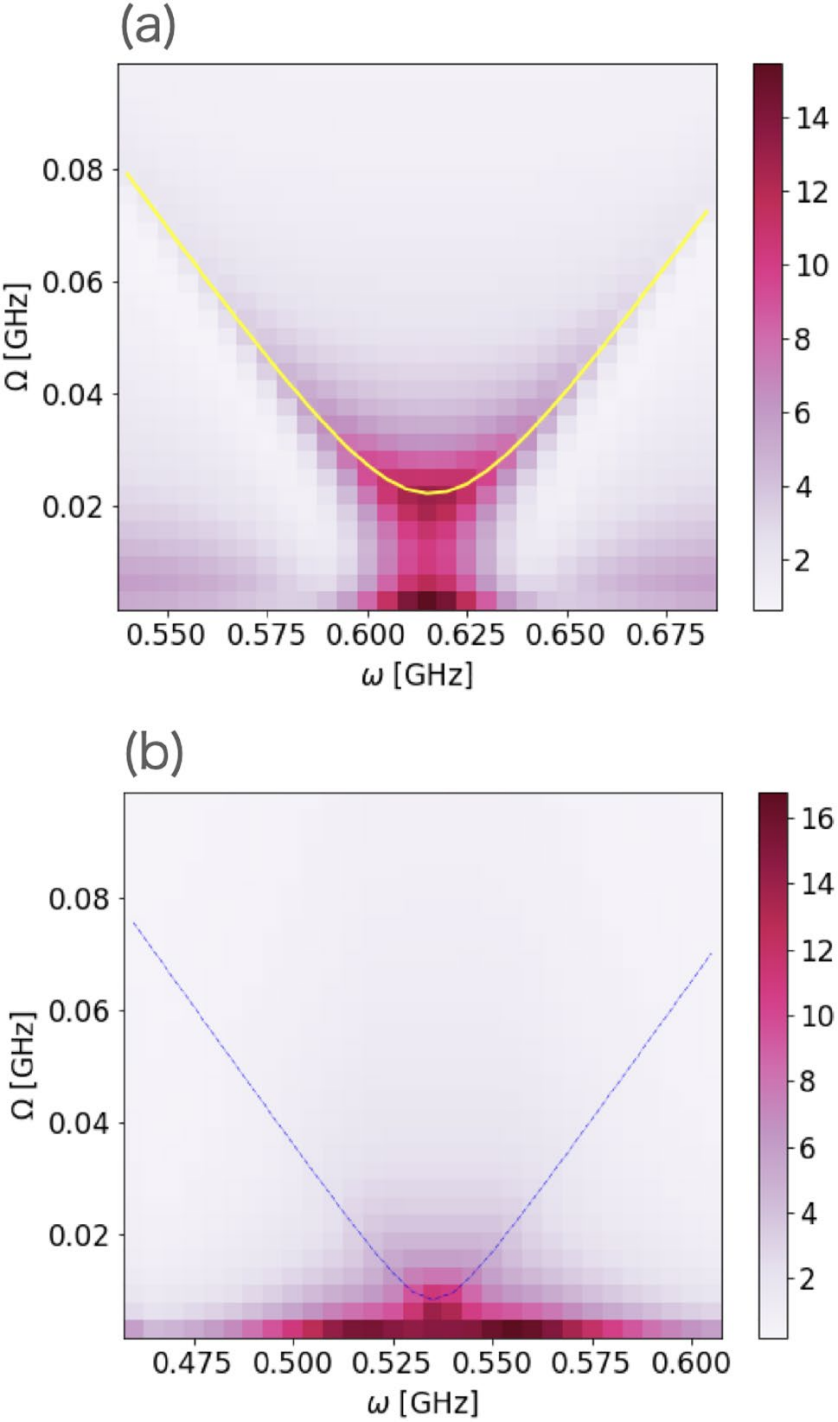
Plot of the power spectrum $P(\omega ,\Omega )$ for $T_{{{{\rm ann}}}} = 100$ for case F (two qubits, incomplete adiabaticity, and decoherence). The horizontal axis represents the angular frequency of the driving field and the vertical axis represents the Fourier frequency. (**a**) We use $s_{1}=0.3$. The yellow line is obtained by fitting Eq. (20) to the plot. (**b**) We use $s_{1}=0.9$. The dotted line represents the exact value obtained by diagonalization.

We plot the estimated transition matrix element and energy gap against $s_{1}$, and we demonstrate that our method is accurate except for two points, $s_{1}=0.3$ and $s_{1}=0.9$ for $T_{\rm ann}=100\ \textrm{ns}$, as shown in Fig. 9. In the former case, as shown in the power spectrum (see Fig. 10a), where $\omega$ is smaller than 0.575 or larger than 0.65, a low-frequency ($\Omega < 0.02$) peak exists, and the height of this peak is greater than that of the target peak at the same $\omega$.

As shown in Eq. (24), strictly speaking, a peak around $\Omega \simeq 0$ should exist in the spectrum, and this peak has a finite width owing to decoherence so that we can observe this in case F. Therefore, if we naively adopt our method described in Eq. (29), we generate an inappropriate $\Omega _{\rm exp}(\omega )$ and obtain incorrect estimated values of the transition matrix element and energy gap.

To identify the target peak in the presence of decoherence and non-adiabatic conditions, we employ a modified approach outlined as follows. Initially, we assess the value of $\Omega$ not only for the highest peak but also for the second- and third-highest peaks at each $\omega$. These pairs of values $(\omega , \Omega )$ then constitute candidates for the data in the estimated function $\Omega _{\rm exp}(\omega )$. Subsequently, we attempt to fit the data using the analytical formula presented in Eq. (20). In the third step, we eliminate data that cannot be adequately fitted by the analytical formula. Finally, we designate data successfully fitting the analytical formula as the target peaks.

In the former case ($s_{1}=0.3$), after using this modified method, the relative error of the transition matrix element (energy gap) is $1.0\%$ $(0.2\%)$, and the ratio to the resolution is 0.07 (0.26). Thus, our modified method is effective for this case, as shown in Fig. 10a.

However, in the latter case ($s_{1}=0.9$), the decoherence is so strong that the target peak nearly disappears, and we cannot identify the target peak anymore, as shown in Fig. 10b.

## Conclusions and discussion

We have proposed an experimental method to assess adiabaticity in QA by evaluating adiabatic conditions. Our approach uses an oscillating field to induce Rabi oscillations, providing insights into the energy gap and the transition matrix element of the time derivative of the Hamiltonian. To validate our method, we performed numerical simulations, considering non-adiabatic transitions and decoherence effects. The results confirm the robustness of our method against these experimentally inevitable problems.

In the main text, we have assumed the adiabaticity to be maintained between $s=0$ and $s=s_{1}$. However, the adiabaticity is not explicitly guaranteed. Here, we discuss $s_{1}$ to be updated systematically by performing our method incrementally. Roughly speaking, at $s_1=s^{(n)}_1$ where *n* denotes the number of current step of iterations, we adopt our method and measure the adiabatic condition. Based on the measurement results, we choose $\Delta s$, and we perform our method at $s_1=s^{(n+1)}_1=s^{(n)}_1+\Delta s$. We can employ the perturbation theory outlined in Supplemental Material to discuss how the ground state varies for *s* around $s=s_1$. As the amplitude $c_{n}(s_{1})$ that is given by Eq. (39) in the Supplemental Material becomes larger, the fidelity between the ground state at $s=s_1+\Delta s$ and that at $s=s_1$ decreases. Based on this, it would be reasonable to maintain $\Delta s$ to satisfy the following condition,

36 $$\begin{aligned} \epsilon = \Delta s^{2}\sum _{n=1}^{\tilde{n}}|c_{n}(s_{1})|^{2}, \end{aligned}$$

where $\epsilon$ denotes a small constant and $\tilde{n}$ denotes an upper bound of the excited states to be considered. We may set certain values of $\epsilon$ and $\tilde{n}$ as threshold. Since we can measure $|c_{n}(s_{1})|$ with our method, we can choose $\Delta s$ to satisfy the threshold value.

More specifically, we adopt the following strategy. First, at $s=0$, we can diagonalize the driver Hamiltonian, and so we can calculate $|c_{n}(0)|^{2}$. This lets us determine $\Delta s$ for the next step using Eq. (36). Second, at $s_1=\Delta s$, we can adopt our method to measure the values of $|c_{n}(s_{1})|$, and we determine $\Delta s$ for the next step using Eq. (36). Finally, we repeat the second step and update $s_1=s^{(n)}_1$ to $s_1=s^{(n+1)}_1=s^{(n)}_1+\Delta s$ until we obtain $s=1$ where *n* denotes the number of interations. Obviously, this approach to update $s_1$ incrementally does not guarantee perfect adiabaticity due to the inherent limitations of the perturbation theory. Further study would be needed to check the validity.

While there have been numerous studies evaluating the performance of quantum annealing in the past, they have typically followed the approach of solving specific problems using various algorithms, as seen in references^40,41^. To quantify the performance such as speed and accuracy, such methodologies often focus on factors like the time required to obtain final results and, when the correct solution is known, the accuracy of the solutions. On the other hand, our method provides a way to measure the adiabatic conditions from the experiments. Our results are useful not only for evaluating the performance of computation but also for checking the adiabatic condition during QA for practical purposes, which is completely different from the previous works^40,41^.

In this perspective, our method may be helpful to determine a better annealing schedule and Hamiltonian form to improve the performance of QA. When a phase transition takes place, the performance of QA is degraded. Some methods have been proposed to address this issue in specific cases^42–44^. To apply these methods, we need to change the annealing schedule and form of the Hamiltonian. However, a potential problem is that we cannot easily find a better annealing scheduling or a better form of the Hamiltonian for general problems if we do not know whether the adiabatic conditions are satisfied. Meanwhile, by using our methods to evaluate the adiabaticity of the dynamics, we could select a better annealing schedule and form of the Hamiltonian when we try to solve practical optimization problems using QA. Further research is needed to check the applicability of this direction, which we leave as an open question.

Here, we examine the validity of the parameters used in our numerical simulations. During the spin lock, the system is in the rotating frame and so we can set $\omega _{1} = 1.0~\textrm{GHz}$ and $\omega _{2} = 1.1~\textrm{GHz}$ as the detuning between the microwave frequency and qubit resonance. The reported coherence time of the superconducting qubit^45^ is much longer than $1/\kappa = 400~\textrm{ns}$ which is used in our simulations. Also, it is possible to realize a coupling strength of $g_{1} = 0.5~\textrm{GHz}$ and $g_{2}=0.3~\textrm{GHz}$ by using inductive coupling between the superconducting qubits^46^. We set the Rabi frequency $\lambda$ to be around tens of $\textrm{MHz}$, which is also available in the current experiment^47^.

We also examine the conditions under which our method can be effectively employed. It is necessary for the success of our method to satisfy the condition (27). If the energy gap $E_{1}-E_{0}$ and coherence time $T_{c}$ remain constant with the number of qubits, the condition (27) can be satisfied even if the number of qubits increases. However, this is not sufficient because there may be many excited states just above the first excited state, which makes it difficult to distinguish the first excited state and the others. Fortunately, for specific circumstances, we could apply our method even if there are many excited states just above the first excited state as we will explain below^48,49^.

**Figure 11:**
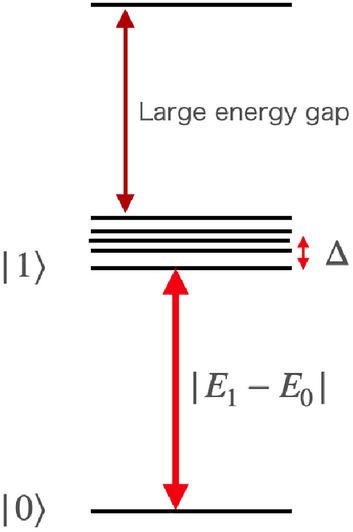
Energy spectrum where many almost degenerate excited states are just above the first excited state. We assume that the power spectrum function of the almost degenerate excited states has a Lorentzian distribution and the linewidth is $\Delta$. Strictly speaking, there are other excited states above these excited states, but we ignore them by assuming that there is a large energy gap.

The Hamiltonian Eq. (18), can be written by

37 $$\begin{aligned} \mathcal {H} = \begin{pmatrix} \omega _{0} &{} c_{1} &{} c_{2} &{} \cdots \\ c_{1}^{*} &{} \omega _{1} &{} &{} \\ c_{2}^{*} &{} &{} \omega _{2} &{} \\ \vdots &{} &{} &{} \ddots \end{pmatrix}, \end{aligned}$$

where $\omega _{i} = (1-r) E_{i}$ and $c_{m}= \lambda {\langle {m|\dot{\mathcal {H}}_{\rm conv}|0}\rangle }/2$. This Hamiltonian is the same as that adopted in Ref.^48^. A spectral density function is defined as $\rho (\omega )= \sum _j |c _j|^2 \delta (\omega -\omega _i)$, and $\rho (\omega )$ is assumed to follow a Lorentzian distribution^48^ (Fig. 11). In this case, we can calculate $\alpha _1(t)={\langle {0|e^{-it\mathcal {H}}|0}\rangle }$ as follows^48^, which corresponds to the probability amplitude at step 5 in our method. We perform the Laplace transform of $\alpha _1(t)$, and obtain,

38 $$\begin{aligned} \mathcal {L}[\alpha _{1}(t)]\propto \frac{s+\frac{1}{2}\Delta }{s^{2}+(\omega _{0}-\frac{i}{2}\Delta ) is + \Omega ^{2}+\frac{i}{2}\Delta \omega _{0}}, \end{aligned}$$

where $\Delta$ is the width of the Lorentz distribution of the spectral distribution,

39 $$\begin{aligned} \Omega ^{2} = \sum _{k}|c_{k}|^{2}, \end{aligned}$$

and $\omega _{0}$ is the center of the distribution which is chosen as zero by adjusting the angular frequency of the external field. Exploiting the inverse Laplace transformation,

40 $$\begin{aligned} \alpha _{1}(t)=e^{-\frac{1}{2}\Delta t}\cos {\sqrt{\Omega ^{2}-\Delta ^{2}/4}t}. \end{aligned}$$

This is a damped oscillation where the angular frequency is $\sqrt{\Omega ^{2}-\Delta ^{2}/4}$ and the decay rate is $\frac{1}{2}\Delta$. By applying our method to this case, we obtain the Rabi frequency $\sqrt{\Omega ^{2}-\Delta ^{2}/4}\simeq \Omega$ and the energy gap $|E_1(s)-E_0(s)| \simeq |E_m(s)-E_0(s)|$ where we assume $\Omega \gg \Delta$ and $|E_1(s)-E_0(s)|\gg \Delta$. We obtain the following

41 $$\begin{aligned} \sum _{m} \frac{|{\langle {m(s)|\dot{\mathcal {H}}(s)|0(s)}\rangle }|^2}{|E_{m}(s)-E_{0}(s)|^{4}}&\simeq \sum _{m} \frac{|{\langle {m(s)|\dot{\mathcal {H}}(s)|0(s)}\rangle }|^2}{|E_{1}(s)-E_{0}(s)|^{4}} \nonumber \\&=\frac{|\Omega |^2}{|E_{1}(s)-E_{0}(s)|^{4}}. \end{aligned}$$

Importantly, by using our method, we can experimentally obtain the value of $\frac{|\Omega |^2}{|E_{1}(s)-E_{0}(s)|^{4}}$, which approximately provides an upper-bound of $\frac{|{\langle {m(s)|\dot{\mathcal {H}}(s)|0(s)}\rangle }|^2}{|E_{m}(s)-E_{0}(s)|^{4}}$. Therefore, if $\frac{|\Omega |^2}{T_\mathrm{{ann}}^2|E_{1}(s)-E_{0}(s)|^{4}}$ is much smaller than 1, the adiabatic condition of $\frac{|{\langle {m(s)|\dot{\mathcal {H}}(s)|0(s)}\rangle }|}{|E_{m}(s)-E_{0}(s)|^{2}T_\mathrm{{ann}}}\ll 1$ should be satisfied. This is how our method to measure the adiabatic condition is useful for a specific circumstance where there are many almost degenerate excited states just above the first excited state.

In QA, the energy gap could approach to zero as we increase the size of the system. In such a case, we will find a highly degenerate spectrum. In our method, we sweep $s_1$ and investigate the adiabatic condition for several values of $s_1$.

Since we sweep the value of $s_1$ from 0 to another value in our method, we can study the adiabatic condition during QA before the energy gap closes. In this case, we will recognize that the energy gap becomes smaller as we increase $s_1$, and this lets us know the existence of the energy gap closing. We could adopt several strategies to enlarge the energy gap in this case. For example, twisted field^50,51^, counterdiabatic term^52,53^, nonstoquastic Hamiltonian^54–56^, inhomogeneous driving magnetic field^57^ may be helpful for such a purpose.

If one of such strategies succeeds, our method will work, and we can investigate the adiabatic conditions during the entire QA process.

Finally, we comment on the adiabatic condition itself. In general, it has not been proved that the condition (1) is sufficient to achieve the adiabaticity. In addition, more sophisticated criteria have been proposed^28^, and it was shown that higher order derivative of the annealing Hamiltonian could affect the adiabaticity. In our numerical examples, we show that the condition (1) actually provides an upper bound of the population of the excited state due to the non-adibatic transitions in Supplemental Material. However, if we need to know the information of the higher order derivative of the Hamiltonian, we can use a modified version of our method. For example, if we are interested in the value of $|{\langle {m|\ddot{H}|0}\rangle }|$, we can replace $\dot{H}(s_{1})$ in Eq. (7) with $\ddot{H}(s_{1})$. We leave a detailed study of this for future work. Secondly, although the conventional adiabatic condition in Eq. (1) is derived from the unitary evolution, it is possible to generalize the adiabatic theorem to open quantum systems^5^. The aim of our method is to know the value of Eq. (1), which is different from the adiabatic condition in the open quantum systems. The extension of our method to the adiabatic condition in open quantum systems is an open question.

### Supplementary Information

Supplementary Information.

## Data Availability

The datasets used and/or analyzed during the current study are available from Y.Mori on reasonable request.
