# Supplementary material for: How to experimentally evaluate the adiabatic condition for quantum annealing
Source: Sci Rep. 2024 Apr 8;14:8177. doi: 10.1038/s41598-024-58286-2 (PMC11001971; doi:10.1038/s41598-024-58286-2)
Supplement: Supplementary file 1 — Supplementary Information. [file 41598_2024_58286_MOESM1_ESM.pdf]

How to experimentally evaluate the adiabatic condition for quantum annealing–Supplementary Material

Yuichiro Mori,[1, *] Shiro Kawabata,[1, 2, †] and Yuichiro Matsuzaki[1, 2, ‡]

[1] *Global Research and Development Center for Business by Quantum-AI Technology (G-QuAT), National Institute of Advanced Industrial Science and Technology (AIST), 1-1-1, Umezono, Tsukuba, Ibaraki 305-8568, Japan*

[2] *NEC-AIST Quantum Technology Cooperative Research Laboratory, National Institute of Advanced Industrial Science and Technology (AIST), Tsukuba, Ibaraki 305-8568, Japan*

I. ADIABATIC THEOREM AND ADIABATIC CONDITION

In this section, we review the adiabatic theorem. We consider a time-dependent Hamiltonian $\mathcal{H}(s)$. For each time s, we denote the eigenstates (called instantaneous eigenstates) obtained by diagonalizing the Hamiltonian $\mathcal{H}(s)$ as $|n(s)\rangle$ and the eigenvalues (called instantaneous energy) as $E_n(s)$.

$$\mathcal{H}(s)\,|n(s)\rangle = E_n(s)\,|n(s)\rangle \tag{1}$$

For any state $|\psi(s)\rangle$, at each time s, the state can be expanded using the instantaneous eigenstates $|n(s)\rangle$ as follows:

$$|\psi(s)\rangle = \sum_n c_n(s) e^{-isT_{\mathrm{ann}}\bar{E}_n(s)}\,|n(s)\rangle\,, \tag{2}$$

where $\bar{E}_n(s)$ is defined by

$$\bar{E}_n(s) = \frac{1}{s}\int_0^s d\sigma E_n(\sigma). \tag{3}$$

Since this $|\psi(s)\rangle$ is a solution of the Schrödinger equation, the state satisfies

$$\begin{aligned} i\frac{d}{ds}\,|\psi(s)\rangle &= i\sum_n \frac{d}{ds}(c_n(s)e^{-isT_{\mathrm{ann}}\bar{E}_n(s)}\,|n(s)\rangle) \\ &= i\sum_n \dot{c}_n(s)e^{-isT_{\mathrm{ann}}\bar{E}_n(s)}\,|n(s)\rangle \\ &\quad + (-i\frac{d}{ds}(sT_{\mathrm{ann}}\bar{E}_n(s)))c_n(s)e^{-isT_{\mathrm{ann}}\bar{E}_n(s)}\,|n(s)\rangle \\ &\quad + c_n(s)e^{-isT_{\mathrm{ann}}\bar{E}_n(s)}\,|\dot{n}(s)\rangle \\ &= \sum_n c_n(s)T_{\mathrm{ann}}E_n(s)e^{-isT_{\mathrm{ann}}\bar{E}_n(s)}\,|n(s)\rangle\,. \end{aligned} \tag{4}$$

Combining Eq. (4) and the orthonormality of the eigenstates, we obtain

$$i\dot{c}_n(s)e^{-isT_{\mathrm{ann}}\bar{E}_n(s)} + i\sum_m c_m(s)e^{-isT_{\mathrm{ann}}\bar{E}_m(s)}\,\langle n(s)|\dot{m}(s)\rangle = 0. \tag{5}$$

Now, we differentiate both sides of Eq. (1) at time s:

$$\dot{\mathcal{H}}(s)\,|n(s)\rangle + \mathcal{H}(s)\,|\dot{n}(s)\rangle = \dot{E}_n(s)\,|n(s)\rangle + E_n(s)\,|\dot{n}(s)\rangle\,. \tag{6}$$

By taking the inner product with $|m(s)\rangle$ again, Eq. (5) becomes

$$\begin{aligned} &\dot{c}_n(s) + \langle n(s)|\dot{n}(s)\rangle\, c_n(s) \\ &= \sum_{m\neq n}\frac{\langle n(s)|\dot{\mathcal{H}}|m(s)\rangle}{E_n(s)-E_m(s)}c_m(s)e^{isT_{\mathrm{ann}}(\bar{E}_n(s)-\bar{E}_m(s))}. \end{aligned} \tag{7}$$

When we ignore the right-hand side of Eq. (7), we can get the adiabatic theorem. Indeed, it is clear that no transitions between the energy levels occur in this time evolution, because the differential equation contains only one variable $c_n(t)$.

To obtain the final state after the time evolution, we integrate both sides of Eq. (7), and we obtain

$$\begin{aligned} c_n(s) - c_n(0) &= -\int_0^s \langle n(\sigma)|\dot{n}(\sigma)\rangle\, c_n(\sigma)d\sigma \\ &+ \int_0^s \sum_{m\neq n}\frac{\langle n(\sigma)|\dot{\mathcal{H}}|m(\sigma)\rangle}{E_n(\sigma)-E_m(\sigma)}c_m(\sigma)e^{iT_{\mathrm{ann}}\sigma(\bar{E}_n(\sigma)-\bar{E}_m(\sigma))}d\sigma. \end{aligned} \tag{8}$$

If necessary, we perform a transformation of the basis $|\tilde{n}(s)\rangle = e^{i\theta(s)}\,|n(s)\rangle$, and $\langle n(s)|\dot{n}(s)\rangle$ can be zero. Thus, Eq. (8) becomes

$$\begin{aligned} &c_n(s) - c_n(0) \\ &= \int_0^s \sum_{m\neq n}\frac{\langle n(\sigma)|\dot{\mathcal{H}}|m(\sigma)\rangle}{E_n(\sigma)-E_m(\sigma)}c_m(\sigma)e^{iT_{\mathrm{ann}}\sigma(\bar{E}_n(\sigma)-\bar{E}_m(\sigma))}d\sigma. \end{aligned} \tag{9}$$

By recursive use of Eq. (9), we obtain a form of $c_n(s)$ as an infinite series. If we use the first-order perturbation, we obtain

$$\begin{aligned} &c_n(s) \\ &\simeq c_n(0) + \sum_{m\neq n} c_m(0)\int_0^s \frac{\langle n(\sigma)|\dot{\mathcal{H}}|m(\sigma)\rangle}{E_n(\sigma)-E_m(\sigma)}e^{iT_{\mathrm{ann}}\sigma(\bar{E}_n(\sigma)-\bar{E}_m(\sigma))}d\sigma \\ &= c_n(0) - i\sum_{m\neq n} c_m(0)\left(A_{mn}(s) - A_{mn}(0) + B_{mn}(s)\right), \end{aligned} \tag{10}$$

* mori-yuichiro.9302@aist.go.jp
† s-kawabata@aist.go.jp
‡ Present E-mail address:ymatsuzaki872@g.chuo-u.ac.jp

where

$$A_{mn}(s) = \frac{\langle n(s)|\dot{\mathcal{H}}|m(s)\rangle}{(E_n(s)-E_m(s))^2} e^{isT_{\rm ann}(\bar{E}_n(s)-\bar{E}_m(s))}, \quad (11)$$

$$B_{mn}(s) = \int_0^s \frac{e^{iT_{\rm ann}\sigma(\bar{E}_n(\sigma)-\bar{E}_m(\sigma))}}{(E_n(\sigma)-E_m(\sigma))} \frac{d}{d\sigma}\left[\frac{\langle n(\sigma)|\dot{\mathcal{H}}|m(\sigma)\rangle}{E_n(\sigma)-E_m(\sigma)}\right] d\sigma. \quad (12)$$

Here, we use

$$\frac{d}{d\sigma}\left[\sigma(\bar{E}_n(\sigma)-\bar{E}_m(\sigma))\right] = (E_n(\sigma)-E_m(\sigma)). \quad (13)$$

When Eq. (**??**) is satisfied, $A_{mn}(s)$ is negligible. In general, if $B_{mn}(s)$ is nonzero, $c_n(s)$ is different from $c_n(0)$ in the first-order perturbation so that the adiabaticity is not always guaranteed by Eq. (**??**) [1, 2]. However, if E_n and E_m are time-independent, $B_{mn}(s)$ corresponds to a Fourier transformation; hence, $B_{mn}(s)$ can be ignored unless the integrating function includes a component whose angular frequency corresponds to the (average) energy gap $\bar{E}_n - \bar{E}_m$. Except for the special non-negligible B_{mn} case, the condition (**??**) makes $c_n(s) = c_n(0)$, and it shows the statement of the adiabatic theorem.

Usually, when we consider QA, the initial states are designated as

$$c_0 = 1, \; c_n = 0 \; (n \neq 0), \quad (14)$$

so that we can finally obtain

$$c_0(s) = 1, \; c_n(s) = -i(A_{0n}(s) - A_{0n}(0)). \quad (15)$$

Furthermore, $c_n(s) \simeq 0$ if the change in the Hamiltonian is sufficiently slow.

II. RABI OSCILLATION

We focused on the characteristics of the dynamics of the system in our proposal. As explained in the main text, our scheme for a single qubit is equivalent to the conventional Rabi oscillation as long as the dynamics at the second and forth steps are adiabatic. Meanwhile, if non-adiabatic transitions occur at the steps, the observed dynamics in our scheme deviates from the conventional Rabi oscillation.

A. Conventional Rabi oscillation

Based on the discussion in the main text, we consider a Rabi oscillation between the states $|k\rangle$ and $|l\rangle$ (for example, the Hamiltonian in Eq. (**??**) corresponds to a case with $k = 0$ and $l = m$). We have

$$\mathcal{H}_{\rm eff} = (1-r)\frac{\Delta}{2}\sigma_z + \frac{\tilde{\lambda}}{2}|k\rangle\langle l| + \frac{\tilde{\lambda}^*}{2}|l\rangle\langle k|, \quad (16)$$

where $\sigma_z = |l\rangle\langle l| - |k\rangle\langle k|$, $\Delta = E_l - E_k$, and $\tilde{\lambda} = \lambda\langle l|\dot{\mathcal{H}}_{\rm QA}|k\rangle$. This coincides with the conventional Hamiltonian to induce the Rabi oscillation, where $\tilde{\lambda}$ denotes the Rabi frequency and $(1-r)\Delta$ denotes the detuning. For this Hamiltonian, the operation $e^{i\theta\sigma_z}$ is adopted for rotation about the z-axis by an appropriate angle θ. Then, the Hamiltonian (16) becomes

$$\mathcal{H}_{\rm eff} = (1-r)\frac{\Delta}{2}\sigma_z + \frac{|\tilde{\lambda}|}{2}\sigma_x, \quad (17)$$

where $\sigma_x = |k\rangle\langle l| + |l\rangle\langle k|$. We can rewrite Eq. (17) with a unitary operator $U_{\rm diag}$ as

$$\mathcal{H}_{\rm eff} = \frac{\tilde{\lambda}'}{2} U^\dagger_{\rm diag}\sigma_x U_{\rm diag}, \quad (18)$$

where $\tilde{\lambda}'$ satisfies

$$\frac{\tilde{\lambda}'}{2} = \sqrt{\left(\frac{|\tilde{\lambda}|}{2}\right)^2 + \left((1-r)\frac{\Delta}{2}\right)^2}. \quad (19)$$

By setting $r = \omega/\Delta$, we obtain $\tilde{\lambda}' = \Omega^{(k,l)}_{\rm ana}(\omega)$ in Eq. (**??**).

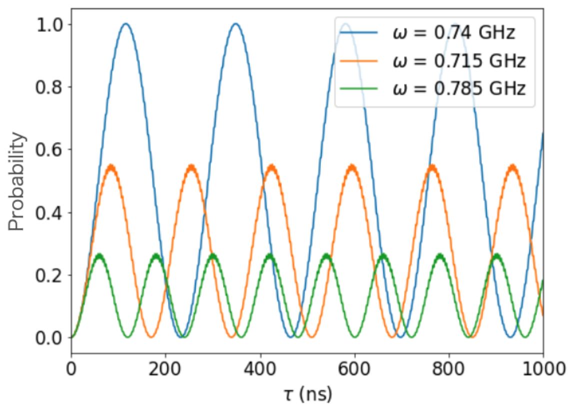

FIG. 1. Plot of the Rabi oscillation. We use a single-qubit Hamiltonian (**??**), with $T_{\rm ann} = 30$ and $t_1 = 0.3\,T_{\rm ann}$. The oscillation period is the longest when the angular frequency ω of the external field coincides with the energy gap $\Delta = 0.74$, indicating that the oscillation period is shorter for small angular frequencies ($\omega = 0.715$) and large angular frequencies ($\omega = 0.785$).

To observe the Rabi oscillations, we calculate the amplitude as follows:

$$\langle l|e^{-it\mathcal{H}_{\rm eff}}|k\rangle = \langle l|U^\dagger_{\rm diag}e^{-it\frac{\tilde{\lambda}'}{2}\sigma_x}U_{\rm diag}|k\rangle,$$
$$= -i\sin\frac{\tilde{\lambda}'}{2}t\,\langle l|U^\dagger_{\rm diag}\sigma_x U_{\rm diag}|k\rangle, \quad (20)$$

using the relations

$$e^{i\theta\sigma_x} = \cos\theta + i\sin\theta\sigma_x, \quad (21)$$
$$\langle l|U^\dagger_{\rm diag}U_{\rm diag}|k\rangle = 0. \quad (22)$$

Finally, we obtain

$$|\langle l|e^{-it\mathcal{H}_{\rm eff}}|k\rangle|^2 = |\langle l|U_{\rm diag}^\dagger \sigma_x U_{\rm diag}|k\rangle|^2 \frac{1-\cos\tilde{\lambda}' t}{2}. \tag{23}$$

Owing to the form of Eq. (19), the minimum value of $\tilde{\lambda}'$ is given by $s=1$; then, the angular frequency of the Rabi oscillation $\tilde{\lambda}'$ is $|\tilde{\lambda}| = \lambda|\langle l|\dot{\mathcal{H}}|k\rangle|$. In this case, $U_{\rm diag} = I$ and the amplitude is maximized. Thus, the Rabi oscillation is a type of resonance phenomenon whose resonant angular frequency is the energy gap Δ.

When the dynamics in the second and fourth steps is adiabatic, both the initial state and the final state are energy eigenstates. Meanwhile, Fig. 1 shows that the Rabi oscillations were obtained numerically using the Hamiltonian induced by Eq. (??) directly, without the approximation described in this section. It is generally a simple sinusoidal curve; however, there are slight fine oscillations when the details are observed.

B. Dynamics with non-adiabatic conditions

We explain the dynamics of our system when the non-adiabatic transitions occur in the second and fourth steps. Here, for simplicity, we assume that RWA is valid. Owing to the non-adiabatic transitions, in general, our prepared state at time t_1 is not an energy eigenstate; it is a superposition state of the energy eigenstates. Similarly, the non-adiabatic transition also occurs from $t_1+\tau$ to $2t_1+\tau$. We will explain that these non-adiabatic transitions cause high-frequency oscillations in our scheme.

In step 3, we prepare a state $|\psi(0)\rangle$ and let this state evolve by the Hamiltonian for time t. The state we obtain at time t denotes $|\psi(t)\rangle$. Combining the unitary evolution in step 4 with the projective measurement performed in step 5, this process can be considered as a projective measurement $|\phi(t)\rangle\langle\phi(t)|$ on the state $|\psi(t)\rangle$.

An overlap between the states is given by $\langle\phi(t)|\psi(t)\rangle = \langle\tilde{\phi}(t)|\tilde{\psi}(t)\rangle$, where we define

$$\langle\tilde{\phi}(t)| = \langle\phi(t)|\, e^{-irt\mathcal{H}_{\rm QA}}. \tag{24}$$

Using RWA, the final transition amplitude is calculated as

$$\begin{aligned}\langle\tilde{\phi}(t)|\tilde{\psi}(t)\rangle &= \langle\tilde{\phi}(t)|e^{-it\mathcal{H}_{\rm eff}}|\tilde{\psi}(0)\rangle\,,\\ &= \langle\phi(t)|e^{-irt\mathcal{H}_{\rm QA}}e^{-it\mathcal{H}_{\rm eff}}|\psi(0)\rangle\,,\end{aligned} \tag{25}$$

where we use the effective Hamiltonian described in Eq. (??). As we prepare a superposition of different energy eigenstates by a non-adiabatic transition and then perform a projective measurement onto another superposition of the energy eigenstates, the difference between the energy eigenvalues affects the oscillation. We will demonstrate this point below.

Let us assume that we are interested in only two states, $|k\rangle$ and $|l\rangle$. In this case, we can approximate the Hamiltonian as $\mathcal{H}_{\rm QA} \simeq \frac{\Delta}{2}\sigma_z = |l\rangle\langle l| - |k\rangle\langle k|$. Furthermore, we can use Eq. (17) for the effective Hamiltonian. The transition amplitude (25) is calculated as

$$\begin{aligned}\langle\phi(t)|e^{-irt\frac{\Delta}{2}\sigma_z}e^{-it\mathcal{H}_{\rm eff}}|\psi(0)\rangle &= \cos\frac{\tilde{\lambda}'}{2}t\,\langle\phi(t)|e^{-irt\frac{\Delta}{2}\sigma_z}|\psi(0)\rangle - i\sin\frac{\tilde{\lambda}'}{2}t\,\langle\phi(t)|e^{-irt\frac{\Delta}{2}\sigma_z}U_{\rm diag}^\dagger\sigma_x U_{\rm diag}|\psi(0)\rangle\,,\\ &= \cos\frac{\tilde{\lambda}'}{2}t\left(\langle\phi(t)|l\rangle\,\langle l|\psi(0)\rangle\,e^{-irt\frac{\Delta}{2}} + \langle\phi(t)|k\rangle\,\langle k|\psi(0)\rangle\,e^{irt\frac{\Delta}{2}}\right)\\ &\quad - i\sin\frac{\tilde{\lambda}'}{2}t\left(\langle\phi(t)|l\rangle\,\langle l|U_{\rm diag}^\dagger\sigma_x U_{\rm diag}|\psi(0)\rangle\,e^{-irt\frac{\Delta}{2}} + \langle\phi(t)|k\rangle\,\langle k|U_{\rm diag}^\dagger\sigma_x U_{\rm diag}|\psi(0)\rangle\,e^{irt\frac{\Delta}{2}}\right).\end{aligned} \tag{26}$$

We can see that the absolute square of Eq. (26) includes five different frequency modes:

$$\begin{aligned}\Omega &= 0,\ \tilde{\lambda}',\ r\Delta-\tilde{\lambda}',\ r\Delta,\ r\Delta+\tilde{\lambda}'.\\ &= 0,\ \tilde{\lambda}',\ \omega-\tilde{\lambda}',\ \omega,\ \omega+\tilde{\lambda}'.\end{aligned} \tag{27}$$

Although we have five peaks, it is easy to specify the target peak for the following reason. As mentioned in the main text, we sweep the frequency range of $0<\Omega\ll\omega$; hence, we observe only a peak $\tilde{\lambda}'$.

III. RESULTS FOR STRONG NON-ADIABATIC TRANSITIONS

In the main text, we considered a case in which the non-adiabatic transition is not relevant. In this section, we investigate the performance of our scheme when we increase the effect of the non-adiabatic transitions in case E.

We plot the spectrum by setting $T_{\rm ann}=3$ and $s_1=0.9$, as shown in Fig. 2 (a). Here, as a visual guide, we plot a blue line corresponding to the analytical curve of Eq. (??), where we use the actual values of $|\langle 1|\dot{\mathcal{H}}|0\rangle|$ and (E_1-E_0), and this is the target peak in the spectrum.

The magnified view is shown in Fig. 2 (b).

We observe unexpected peaks at frequencies of around $\Omega = 0.38$ in the spectrum, and their height is more significant than that of the target peak. Therefore, if we naively adopt our method described in Eq. (??), we obtain incorrect estimated values of the transition matrix element and energy gap.

To understand the origin of these unexpected peaks, we perform analytical calculations in order to obtain resonant frequencies in the spectrum with non-adiabatic transitions in Appendix II B. Although the peaks at $\Omega = \omega - \Omega_{\rm ana}^{(0,1)}(\omega),\ \omega,\ \omega + \Omega_{\rm ana}^{(0,1)}(\omega)$ should exist, we could not observe them owing to the restricted range of Ω in the spectrum, as mentioned in Appendix II B. Meanwhile, if there is a non-negligible population of the second excited state induced by the non-adiabatic transitions, we can observe peaks at frequencies of $\Omega = \omega - \Omega_{\rm ana}^{(1,2)}(\omega),\ \omega,\ \omega + \Omega_{\rm ana}^{(1,2)}(\omega)$. To see these points, we plot the spectrum in Fig. 3, and we actually observe three such peaks. Moreover, when ω is far from the energy difference $(E_2 - E_1)$, the peak at a frequency of $\omega - \Omega_{\rm ana}^{(1,2)}(\omega)$ asymptotically approaches the energy difference $(E_2 - E_1)$, and this is the origin of the highest peaks in Fig. 2(a). The frequency of $\omega - \Omega_{\rm ana}^{(1,2)}(\omega)$ is much lower than $\omega - \Omega_{\rm ana}^{(0,1)}(\omega),\ \omega,\ \omega + \Omega_{\rm ana}^{(0,1)}(\omega)$; therefore, we observe this peak even if we restrict the range of Ω in the spectrum.

Even when we observe peaks other than the target peaks, there is a way to estimate the transition matrix element and energy gap. As mentioned in the main text (in Section ??), we adopt a modified method to identify the target peak, which is useful for this case as well. The positions of the target peak are expected to be fitted by the analytical formula in Eq. (??). Thus, if we fail to fit the peaks, we can guess that such peaks do not correspond to the peak from the Rabi oscillation. Indeed, the peak $\Omega \simeq 0.38$ cannot be well fitted by Eq. (??). Meanwhile, if we focus on the peaks with frequencies of around $\Omega = 0.01$, as shown in Fig. 2 (b), we can fit these peaks by the analytical formula; hence, we can accurately estimate the transition matrix element and energy gap.

Furthermore, we plot the spectrum by setting $T_{\rm ann} = 3$ and $s_1 = 0.7$ in Fig. 4 (a). Here, as a visual guide, we plot a yellow line corresponding to the analytical curve of Eq. (??), which are the target peaks. Here, the target peaks as well as other peaks are observed. These come from higher-order perturbations, which can be observed for a larger Rabi frequency (see Appendix IV). We can distinguish these secondary peaks from the target peaks as follows.

First, the secondary peaks are usually smaller than the target peaks. As shown in Fig. 4, except for a few points, the target peaks are the highest in this frequency region. Second, from the fitting results by Eq. (??), we can distinguish the target peaks from the secondary peaks (for example, the slope of the target peaks is given by $\frac{d\Omega_{\rm ana}(\omega)}{d\omega} \simeq 1$ for large ω, while the slope of the secondary

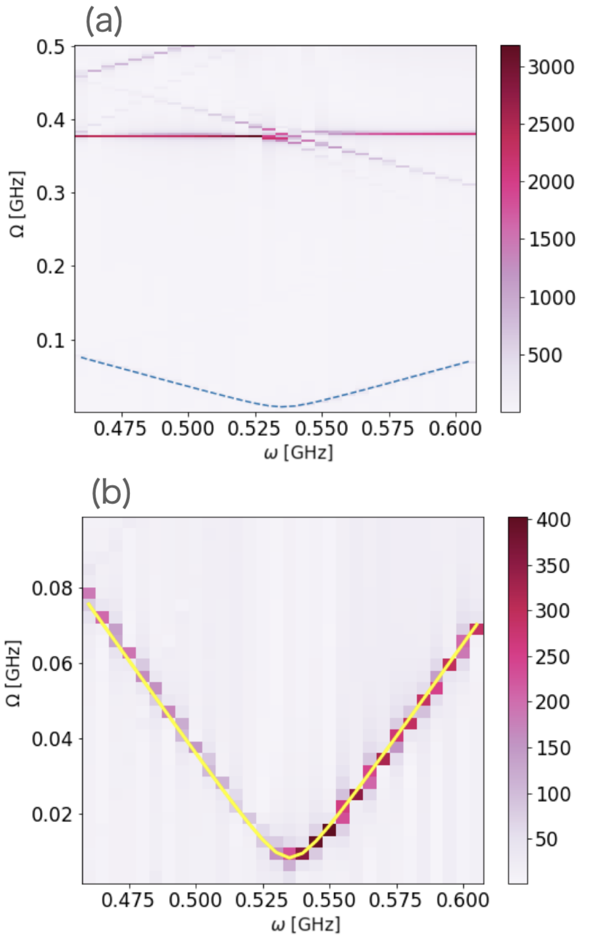

FIG. 2. In case E, we plot the power spectrum $P(\omega, \Omega)$, where we set $T_{\rm ann} = 3$ and $t_1/T_{\rm ann} = 0.9$ in (a). The magnified view is shown in (b). The blue and yellow lines represent the target peaks obtained by diagonalization. The peak we want is seen at the correct position when Ω is enlarged in the small region; however, it is lower than the high-frequency peak around $\Omega = 0.38$.

peaks is twice as large). Third, the λ-dependence of the peak height is different. The height of the target peaks scales as λ^2, while that of the secondary peaks scales as λ^4, and this lets us identify the target peaks by sweeping λ. As shown in Fig. 4, we plot the spectrum by selecting a smaller λ, and we show that the secondary peak becomes nearly invisible compared to the target peak.

IV. PERTURBATIVE APPROACH OF THIS METHOD

In the main text, we used RWA, and the Hamiltonian was effectively transformed into a simple two-dimensional one. When we consider higher-order perturbations, there can be other peaks without the $|E_1 - E_0| = \omega$ condition.

We calculate the transition amplitude $\langle f|U(t,0)|i\rangle$, where $U(t,0)$ is a unitary operator expressing the time

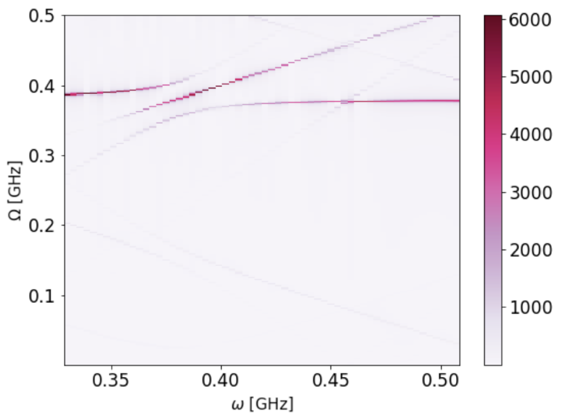

FIG. 3. Plot of the spectrum to focus on the peak owing to a transition from the first excited state to the second excited stat, in case E. We observe three peaks around $\Omega \simeq \omega \simeq 0.38$. These correspond to frequencies of $\Omega = \omega - \Omega_{\rm ana}^{(1,2)}(\omega)$, ω, $\omega + \Omega_{\rm ana}^{(1,2)}(\omega)$.

evolution from time 0 to time t, and $|i\rangle$ and $|f\rangle$ are the initial state and the final state, respectively.

First, we describe the Hamiltonian of Eq. (**??**) in the so-called interaction picture:

$$\tilde{\mathcal{H}}(t) = \lambda e^{it\mathcal{H}_{\rm QA}} \dot{\mathcal{H}}_{\rm QA} e^{-it\mathcal{H}_{\rm QA}} \cos\omega t. \tag{28}$$

Then, the transition amplitude is given by

$$\begin{aligned}\langle f|U(t,0)|i\rangle &= \langle f|i\rangle + (-i)\int_0^t d\tau\, \langle \tilde{f}|\tilde{\mathcal{H}}(\tau)|\tilde{i}\rangle \\ &+ (-i)^2 \int_0^t d\tau_1 \int_0^{\tau_1} d\tau_2\, \langle \tilde{f}|\tilde{\mathcal{H}}(\tau_1)\tilde{\mathcal{H}}(\tau_2)|\tilde{i}\rangle + \cdots,\end{aligned} \tag{29}$$

where the tilde symbol implies that the states are in the interaction picture. As the Schrödinger picture coincides with the interaction picture at $t = 0$, we have $|\tilde{i}\rangle = |i\rangle$. We assume that λ is small, and we calculate the transition amplitude up to the second order of the perturbation theory.

In our method, we assume that the dynamics is adiabatic in the second and fourth steps. In this case, we can set $|i\rangle = |0\rangle$ and $|f\rangle = |m\rangle$, and we evaluate the quantity of $p_{0,m}(t) = |\langle m|U(t,0)|0\rangle|^2$.

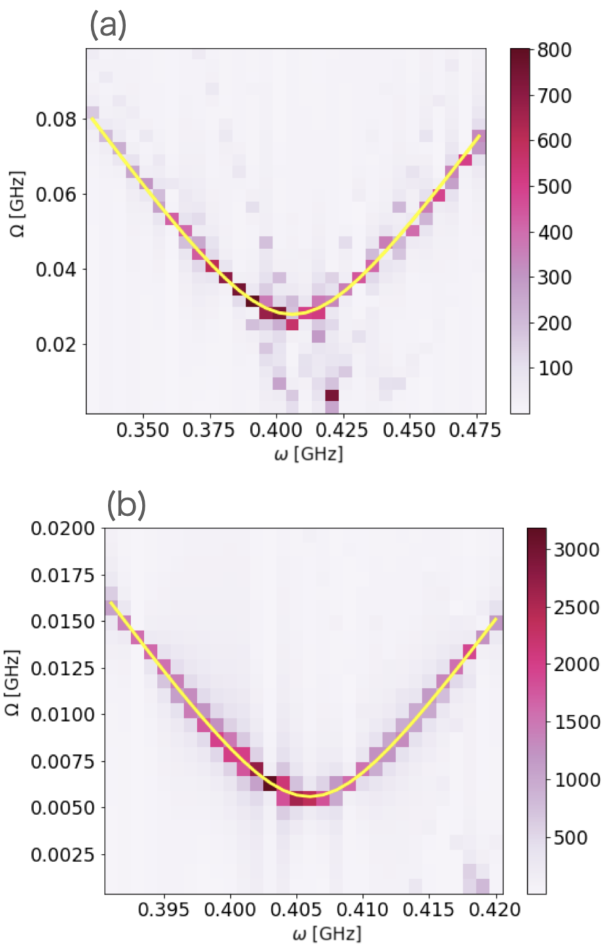

FIG. 4. Plot of the power spectrum in case E with $T_{\rm ann} = 3$ and $t_1/T_{\rm ann} = 0.7$. Here, as a visual guide, we plot a yellow line corresponding to the analytical curve of Eq. (**??**), which are the target peaks. (a) We select $\lambda/T_{\rm ann} = 0.05$. We observe the target peaks as well as the peaks due to higher-order perturbations. (b) We select $\lambda/T_{\rm ann} = 0.01$. Compared to the case with $\lambda/T_{\rm ann} = 0.05$, the peaks caused by higher-order perturbations are smaller.

A. First-order perturbation and the Rabi oscillation

Let us consider the first order of the perturbative term in Eq. (29) as follows:

$$\begin{aligned}
&\int_0^t d\tau \, \langle \tilde{m}|\tilde{\mathcal{H}}(\tau)|\tilde{0}\rangle \\
&= \lambda \int_0^t d\tau \, \langle m|e^{-it\mathcal{H}_{\mathrm{QA}}} e^{i\tau\mathcal{H}_{\mathrm{QA}}} \dot{\mathcal{H}}_{\mathrm{QA}} e^{-i\tau\mathcal{H}_{\mathrm{QA}}}|0\rangle \cos\omega\tau \\
&= \lambda \langle m|\dot{\mathcal{H}}_{\mathrm{QA}}|0\rangle \int_0^t d\tau e^{-iE_m t + i(E_m - E_0)\tau} \cos\omega\tau \\
&= \frac{\lambda}{2} \langle m|\dot{\mathcal{H}}_{\mathrm{QA}}|0\rangle \, e^{-iE_m t} \\
&\quad \cdot \left(\frac{e^{i(E_m - E_0 + \omega)t} - 1}{i(E_m - E_0 + \omega)} + \frac{e^{i(E_m - E_0 - \omega)t} - 1}{i(E_m - E_0 - \omega)} \right). \quad (30)
\end{aligned}$$

The absolute square of Eq. (30) includes terms with frequencies of 2ω, $E_m - E_0 \pm \omega$. This result is consistent with the analytical result in Eq. (??) in the limit of small λ, which is used to predict the resonance at $\Omega = |E_m - E_0 - \omega|$.

B. Second-order perturbation

Let us consider three energy eigenstates of $\mathcal{H}_{\mathrm{QA}}$ as $|0\rangle, |I\rangle, |m\rangle$, and we assume that $|i\rangle = |0\rangle$ and $|f\rangle = |m\rangle$. The third term of the right-hand side of Eq. (29) is given by

$$\begin{aligned}
&(-i)^2\lambda^2 \int_0^t dt_1 \int_0^{t_1} dt_2 \sum_i \langle m|\dot{\mathcal{H}}_{\mathrm{QA}}|i\rangle \langle i|\dot{\mathcal{H}}_{\mathrm{QA}}|0\rangle \\
&\quad \cdot e^{-iE_m t + i(E_m - E_i)t_1} e^{i(E_i - E_0)t_2} \cos\omega t_1 \cos\omega t_2, \quad (31) \\
&= (-i)^2\lambda^2 \langle m|\dot{\mathcal{H}}_{\mathrm{QA}}|I\rangle \langle I|\dot{\mathcal{H}}_{\mathrm{QA}}|0\rangle \\
&\quad \times \frac{e^{i(E_m - E_0 - 2\omega)t} - 1}{(E_I - E_0 - \omega)(E_m - E_0 - 2\omega)} \\
&\quad + \cdots . \quad (32)
\end{aligned}$$

The absolute square of this amplitude includes various modes; however, one of them is $E_m - E_0 - 2\omega$. Thus, the probability function $p_{0,m}(\tau)$ includes an oscillation with a frequency of $\Omega = 2(\omega - (E_m - E_0)/2)$. Importantly, for this frequency, we have $\frac{d\Omega}{d\omega} = 2$, while we have $\frac{d\Omega_{\mathrm{ana}}}{d\omega} = 1$ for our analytical formula in Eq. (??).

V. ON THE VALIDITY OF THE ADIABATIC CONDITION (??)

The adiabatic condition (??) is believed to be related to success of quantum annealing but it is not proven to be sufficient. The adiabatic condition Eq. (??) comes from the equation (11), and the final transition amplitude to the excited states is approximately given by (15). It is worth mentioning that, since a perturbative theory is used, the Eq. (15) is not exact. Although we cannot show the validity of the Eq. (15) for any quantum annealing, we can show that the Eq. (15) actually provides us with an upperbound of the population of the excited states for our numerical examples in section ??. According to Eq. (15), the population of the n-th excited state is evaluated by

$$\begin{aligned}
|c_n(s)|^2 &= |A_{0n}(s) - A_{0n}(0)|^2 \\
&\leq \left(\max_{0\leq\sigma\leq s} [|A_{0n}(\sigma)|] + |A_{0n}(0)| \right)^2 =: p_n(s). \quad (33)
\end{aligned}$$

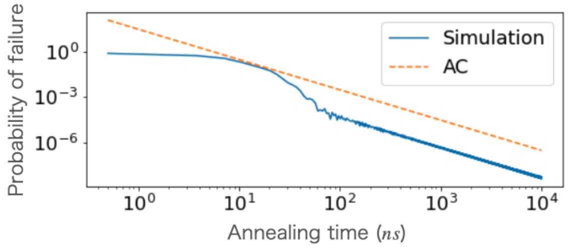

FIG. 5. Plot of the population of being one of the excited states (solid line) and sum of $p_n(1)$ (Dotted line) against the annealing time T_{ann} for $n = 1, 2, 3$. For the large T_{ann}, we confirm that $p_n(1)$ actually gives an upper bound of the population in these cases.

We perform a numerical simulation of the quantum annealing defined by (??) with the two qubit model (??), and calculate the populations of the first, the second, and the third excited states at the time $s = 1$. Then, we plot the dependence of these populations on the annealing time T_{ann}. (see Fig. 5)

Fig. 5 shows that sum of p_n defined by (33) is actually larger than the populations obtained by the quantum annealing for the large T_{ann}. Also, we confirm that the populations are inversely proportional to the square of the annealing time T_{ann} in the limit of the large T_{ann}. If T_{ann} is small, the approximation used in (10) is violated and p_n does not provide the upper bound of the population. Thus, the adiabatic condition we used (??) is expected to be valid for the large T_{ann}.

VI. NUMERICAL RESULTS FOR LARGER SYSTEMS

To establish the applicability of our method for larger systems, we adopt a nine-qubit system with the following

TABLE I. Parameters of the nine-qubit simulation.

h_1	h_2	h_3	h_4	h_5	h_6	h_7	h_8	h_9
1.00	0.10	0.75	0.60	1.10	1.30	0.30	0.26	1.42
$J_{1,2}$	$J_{2,3}$	$J_{3,4}$	$J_{4,5}$	$J_{5,6}$	$J_{6,7}$	$J_{7,8}$	$J_{8,9}$	$J_{9,1}$
-0.91	-0.58	-1.04	-0.95	-0.93	-0.98	-0.75	-0.88	-1.01

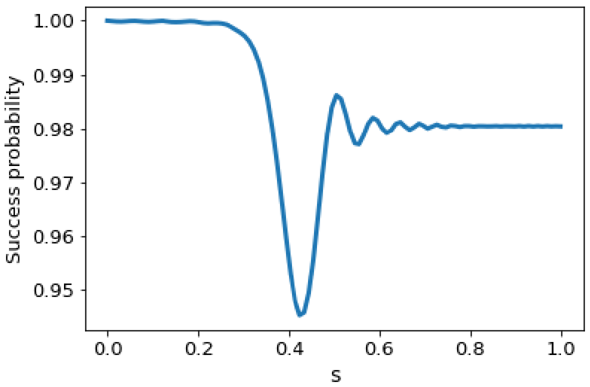

FIG. 6. Success probability of our nine-qubit annealing process with respect to s. Around $s = 0.4$, success probability is suddenly worsen.

Hamiltonian.

$$\mathcal{H}_{\rm D} = \sum_{i=1}^{9} h_i \sigma_z^i,$$
$$\mathcal{H}_{\rm P} = \left(\sum_{i=1}^{8} J_{i,i+1}\sigma_x^i \sigma_x^{i+1}\right) + J_{9,1}\sigma_x^9\sigma_x^1, \quad (34)$$

where σ_x^i (σ_z^i) is the Pauli X (Z) matrix acting on i-th qubit, $J_{i,i+1}$ is a coupling strength between nearest neighbor qubits, h_i is a strength of the external field. Actual values of these parameters are shown in Table I.

Let us explain how we can apply our method to this system. During the implementation of QA with the driver Hamiltonian and problem Hamiltonian described above, a notable observation emerges where the success probability suddenly decreases at a specific point in the process. (see Fig. 6). We apply our method to measure the adiabatic condition at the point, and we obtain a power spectrum depicted as Fig. 7. As depicted in Fig. 7, while the spectrum displays numerous curves, the curve corresponding to the desired signal is discernible, and so we can fit this by using the analytical expression of Eq. (**??**). Therefore, we can estimate the value of the right-hand side of the Eq. (**??**) from the spectrum, and this information is valuable when we try to optimize the schedule of QA to maximize the success probability.

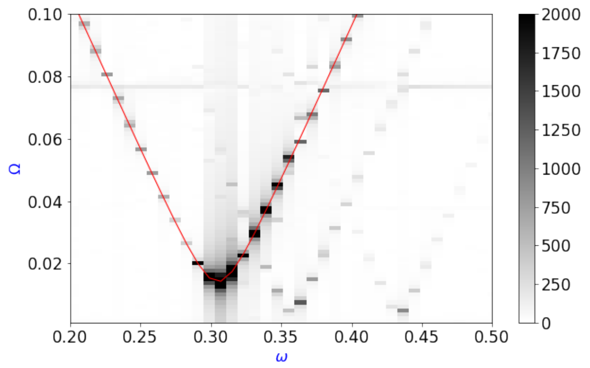

FIG. 7. Power spectrum for measuring of $E_4 - E_0$ and $\langle 4|\dot{H}|0\rangle$ with our method. The brown curve shows the analytically expected curve $\Omega_{\rm ana}(\omega)$ in Eq. (**??**) with diagonalization of the Hamiltonian Eq. (34). We select $\lambda/T_{\rm ann} = 0.01$ in this simulation.

VII. PERTURBATION THEORY AND UPDATE OF s_1

We expect the adiabatic condition at $s = s_1 + \Delta s$ using the perturbation theory and a number of quantities obtained at $s = s_1$. We expand the Hamiltonian $\mathcal{H}(s)$ around $s = s_1$ as,

$$\mathcal{H}(s_1 + \Delta s) = \mathcal{H}(s_1) + \Delta s \dot{\mathcal{H}}(s_1) + o(\Delta s), \quad (35)$$

and we evaluate the eigenvalues and eigenstates of the Hamiltonian $\mathcal{H}(s_1 + \Delta s)$ using the perturbation theory with considering $\mathcal{H}(s_1)$ as the unperturbed Hamiltonian and $\Delta s \dot{\mathcal{H}}(s_1)$ as the perturbation Hamiltonian. Then, we obtain,

$$E_0(s_1 + \Delta s) = E_0(s_1) + \Delta s \Delta E_0(s_1) + o(\Delta s), \quad (36)$$
$$|0(s_1 + \Delta s)\rangle = |0(s_1)\rangle + \Delta s \sum_{n\neq 0,} c_n(s_1)\,|n(s_1)\rangle + o(\Delta s), \quad (37)$$

where $\Delta E_0(s_1)$ and $c_n(\Delta s)$ are given by,

$$\Delta E_0(s_1) = \langle 0(s_1)|\dot{\mathcal{H}}(s_1)|0(s_1)\rangle\,, \quad (38)$$
$$c_n(s_1) = \frac{\langle n(s_1)|\dot{\mathcal{H}}(s_1)|0(s_1)\rangle}{E_n(s_1) - E_0(s_1)}. \quad (39)$$

The absolute value of $c_n(s_1)$ can be evaluated with our method.

[1] M. Amin, Consistency of the adiabatic theorem, Physical Review Letters **102**, 220401 (2009).

[2] A. Dodin and P. Brumer, Generalized adiabatic theorems: Quantum systems driven by modulated time-

varying fields, PRX Quantum **2**, 030302 (2021).
